# Nutrient‐, Food‐Group‐, and Food‐Item‐Level Dietary Signatures of Psoriasis Comorbidity Among Adults With Anxiety Disorder: An Explainable Machine Learning Study

**DOI:** 10.1002/fsn3.72482

**Published:** 2026-10-21

**Authors:** Canni Gao, Xinxin Duan, Mei Luan, Weijie Huang, Guangdong Wang, Zhihong Xu

**Affiliations:** ^1^ The First Affiliated Hospital of Xi'an Jiaotong University Xi'an China; ^2^ Department of Anesthesiology (Xiamen Anesthesia Quality Control Center), The First Affiliated Hospital of Xiamen University, School of Medicine Xiamen University Xiamen China

**Keywords:** anxiety, dietary patterns, machine learning, predictive modeling, psoriasis, SHAP

## Abstract

Psoriasis and anxiety disorders frequently co‐occur, yet the dietary features associated with this comorbidity remain poorly characterized. This cross‐sectional study compared nutrient‐, food‐group‐, and food‐item‐level dietary representations among adults with medication‐defined anxiety disorder using linked National Health and Nutrition Examination Survey (NHANES), Food Patterns Equivalents Database/MyPyramid Equivalents Database (FPED/MPED), and What We Eat in America (WWEIA) data. Three analytic datasets were constructed at the nutrient level (*n* = 1016; 37 psoriasis‐comorbidity cases), food‐group level (*n* = 1215; 46 cases), and food‐item level (*n* = 1257; 46 cases). Each dataset was divided using a fixed stratified 7:3 training‐validation split. Correlation filtering, shadow‐feature selection, scaling, class‐imbalance handling, and hyperparameter tuning were confined to the training workflow, with five‐fold stratified cross‐validation and an untouched validation set. Seven models, including conventional multivariable logistic regression, were compared. In locked validation, the nutrient‐level logistic regression model showed the strongest validation signal (area under the receiver operating characteristic curve [AUC‐ROC] 0.645, 95% confidence interval [CI] 0.464–0.811; area under the precision‐recall curve [AUC‐PR] 0.063; Brier score 0.176). The food‐group logistic regression model (AUC‐ROC 0.396, 95% CI 0.256–0.539) and the food‐item extreme gradient boosting (XGBoost) model (AUC‐ROC 0.401, 95% CI 0.273–0.537) provided more limited validation signals in the present sample. SHapley Additive exPlanations (SHAP) analyses of the cross‐validation‐selected models identified niacin, carbohydrate intake, β‐cryptoxanthin, fat‐related variables, hypertension, body mass index, and selected food categories as influential model features. These contributions are best interpreted as model‐derived signals that prioritize candidate dietary and clinical features for further study. Together, the findings highlight nutrient‐level dietary information as the most promising representation for subsequent development of nutrition‐informed risk assessment in adults with anxiety. Larger prospective cohorts and external validation may further strengthen the clinical applicability of these signals.

## Graphical abstract

Psoriasis frequently coexists with anxiety disorders, yet the dietary signatures associated with this comorbidity remain insufficiently characterized. In this study, linked NHANES, MPED, and WWEIA databases were used to construct nutrient‐, food‐group‐, and food‐item‐level dietary representations among adults with medication‐defined anxiety disorder. Multiple machine‐learning models were developed and evaluated under a leakage‐controlled validation framework, with SHAP analysis applied for model interpretation. Nutrient‐level dietary information showed the strongest validation signal, while key candidate features including niacin, carbohydrate intake, β‐cryptoxanthin, fat‐related variables, body mass index, and selected food categories were identified. This explainable multi‐level dietary framework provides insights into potential nutritional signatures of psoriasis comorbidity and supports future investigations in larger prospective cohorts.

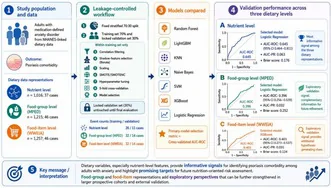

## 1 Introduction

Psoriasis is a systemic, immune‐mediated inflammatory disease, whereas anxiety disorders are primarily characterized by persistent worry, fear, and tension (Guo et al. 2023; Leichsenring et al. 2023). Accumulating clinical and epidemiological evidence suggests a bidirectional relationship between these two conditions: patients with psoriasis frequently exhibit anxiety symptoms (Jalenques et al. 2022), and conversely, an anxious state is associated with an elevated risk of developing psoriasis (Bai and Ma 2025). This comorbidity is thought to involve dysregulation of the “psycho‐neuro‐endocrine‐skin” axis. Specifically, anxiety can activate the hypothalamic–pituitary–adrenal (HPA) axis and the sympathetic nervous system, promoting the release of pro‐inflammatory mediators such as tumor necrosis factor‐α (TNF‐α), interleukin‐6 (IL‐6), and interleukin‐17 (IL‐17), which in turn exacerbate cutaneous inflammation and the abnormal proliferation of keratinocytes (Liu and Chang 2024). Conversely, the visible skin lesions, chronic pruritus, and pain associated with psoriasis can further induce or worsen anxiety through social stigmatization and diminished quality of life (Timis et al. 2023). Consequently, the psoriasis–anxiety comorbidity has emerged as a critical focus within the interdisciplinary field of dermatology and psychiatry (Henderson et al. 2023).

Existing research indicates that the comorbidity of psoriasis and anxiety shares a common biological foundation, including oxidative stress imbalance, inflammatory amplification, and dysfunction of the gut–brain–skin axis (Goel et al. 2025; Kim and Hong 2026). As a significant modifiable exposure, diet may contribute to this comorbid process by modulating systemic inflammation, oxidative stress, immunometabolism, and the gut microbiota (Hawkins et al. 2025; Pachauri and Sharma 2025). Evidence suggests that a higher dietary inflammatory potential and greater consumption of ultra‐processed foods are associated with an increased risk of anxiety symptoms, whereas Mediterranean diet interventions may improve psoriasis severity (Chen et al. 2021; Peng et al. 2025; Perez‐Bootello et al. 2025). However, previous studies have largely focused on either psoriasis or anxiety as a single outcome and have typically relied on a single level of dietary granularity. Such approaches struggle to fully capture the high‐dimensional features and nonlinear associations inherent in real‐world dietary exposures.

We therefore evaluated whether nutrient‐, food‐group‐, and food‐item‐level dietary representations could capture dietary signatures associated with psoriasis comorbidity among adults with medication‐defined anxiety disorder. Linked NHANES, Food Patterns Equivalents Database/MyPyramid Equivalents Database, and WWEIA data were used to construct three complementary dietary representations and benchmark multiple prediction models, with SHAP used for model interpretation. The analysis focused on identifying dietary features associated with model‐predicted psoriasis comorbidity within a multi‐level, explainable framework.

## 2 Methods

### 2.1 Data Sources, Participants, and Study Design

This study is a cross‐sectional analysis based on the National Health and Nutrition Examination Survey (NHANES). Data were derived from the 2005–2006 and 2009–2014 survey cycles of NHANES, linked with the MyPyramid Equivalents Database (MPED) and What We Eat In America (WWEIA). To characterize dietary exposures at different levels of granularity, we constructed three parallel dietary representation systems: dietary micronutrients/macronutrients (nutrient level), food‐group equivalents, and food‐item categories. All three databases can be linked to NHANES demographic and health information via U.S. Department of Agriculture (USDA)/NHANES food codes or participant identifiers, thereby generating an individual‐level analytic dataset.

We initially identified 40,816 participants from the selected survey cycles. We applied stringent exclusion criteria and derived separate analytic samples for the three dietary representations. In the NHANES analytic pipeline, 39,800 participants were excluded due to missing anxiety disorder information or absence of anxiety disorder (*n* = 38,834), missing psoriasis information (*n* = 359), missing dietary micronutrient data (*n* = 381), or missing covariates (education, poverty–income ratio [PIR], body mass index [BMI], hypertension, diabetes, or alcohol use; *n* = 226), yielding a final NHANES analytic sample of 1016 participants (Figure 1). For the MPED‐linked dataset, 39,601 participants were excluded due to missing anxiety disorder information or absence of anxiety disorder (*n* = 38,834), missing psoriasis information (*n* = 359), missing food‐group equivalent data (e.g., meat; *n* = 161), or missing covariates (education, PIR, BMI, hypertension, diabetes, or alcohol use; *n* = 247), resulting in a final MPED analytic sample of 1215 participants (Figure 1). In the WWEIA‐linked dataset, 39,559 participants were excluded due to missing anxiety disorder information or absence of anxiety disorder (*n* = 38,834), missing psoriasis information (*n* = 359), missing WWEIA database information (*n* = 2), or missing covariates (education, PIR, BMI, hypertension, diabetes, or alcohol use; *n* = 364), leaving 1257 participants in the final WWEIA analytic sample (Figure 1).

**FIGURE 1:**
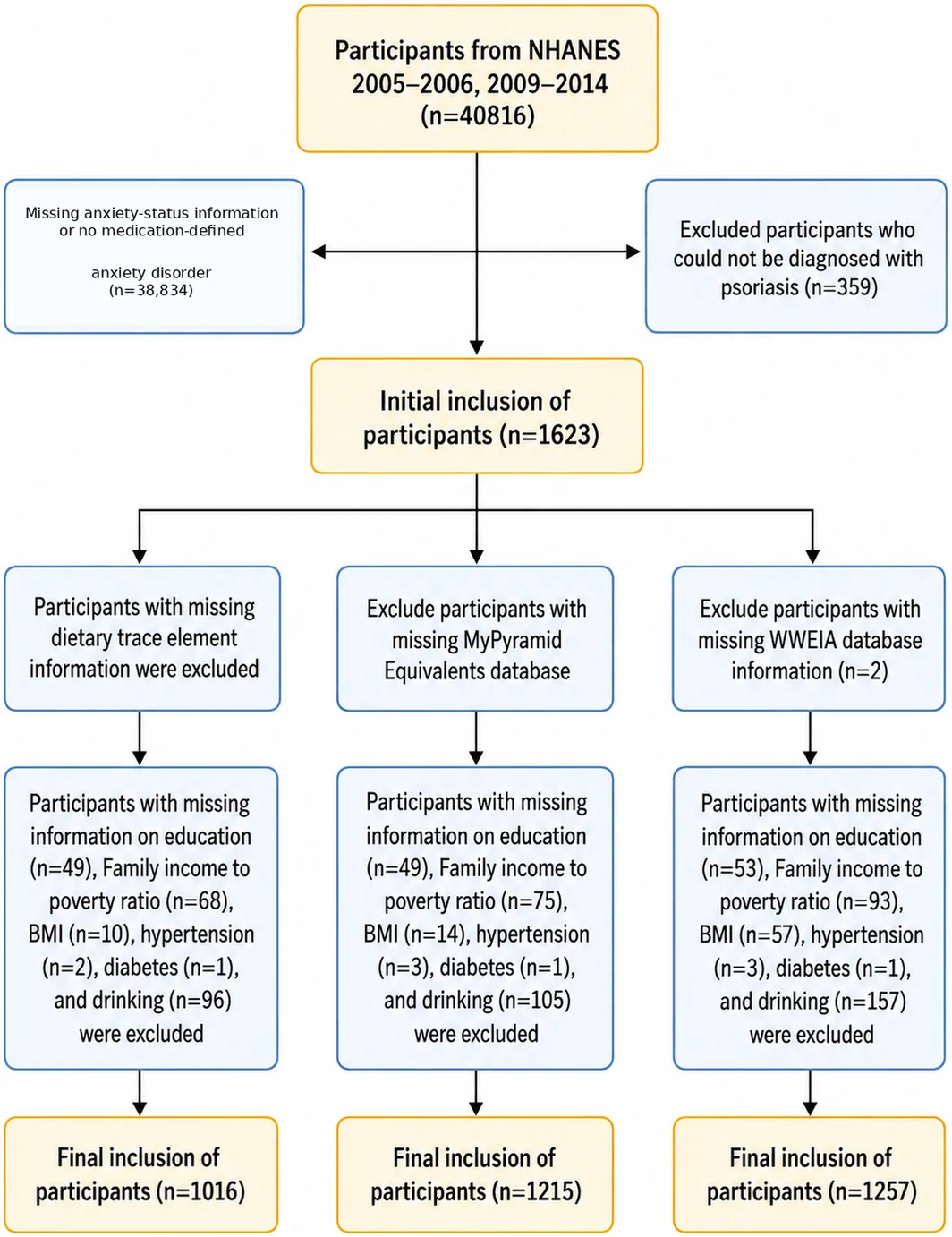
Participant selection flowchart for the NHANES, MPED, and WWEIA analytic datasets. The flowchart summarizes participant screening, sequential exclusion criteria, and the final analytic samples for the nutrient‐level, MPED food‐group, and WWEIA food‐item datasets.

### 2.2 Dietary Predictors, Outcome, and Covariates

Dietary and nutritional exposures were assessed using three complementary representations. Nutrient intakes and food‐group equivalents used in the nutrient‐level and MPED analyses were derived from the first 24‐h dietary recall (Day 1), which was conducted in person at the Mobile Examination Center (MEC) using the Automated Multiple‐Pass Method (AMPM) (Moshfegh et al. 2008). To characterize daily food‐group intake, the USDA MyPyramid Equivalents Database (MPED) was linked to NHANES via USDA food codes to compute daily food‐group equivalent servings, enabling evaluation of gaps between observed intake and dietary recommendations. In addition, individual‐level food intake was assessed using the USDA What We Eat in America (WWEIA) component, which provides standardized 24‐h recall data with detailed food and beverage types, amounts, and consumption context; leveraging USDA standardized food and nutrient coding, WWEIA was accurately linked to NHANES demographic, examination, and laboratory data to estimate individual‐level daily intakes of foods and nutrients for surveillance and epidemiologic research.

The primary outcome of this study was anxiety disorder–psoriasis comorbidity. Anxiety disorder status was ascertained using the NHANES Prescription Medications questionnaire, which summarizes the types and number of prescription drugs taken in the past 30 days (Kantor et al. 2015). Participants were classified as having anxiety disorder if they reported using any medication for “Anxiety disorder” (ICD F41.9) within the past 30 days. Psoriasis was determined by self‐report: participants who answered “yes” to the question “Have you ever been told by a health care provider that you have psoriasis?” were considered to have psoriasis (Kurd and Gelfand 2009). Participants with both anxiety disorder and psoriasis were considered to have anxiety disorder–psoriasis comorbidity.

Covariates included sociodemographic and lifestyle factors: age, sex, race/ethnicity (Mexican American, Other Hispanic, Non‐Hispanic White, Non‐Hispanic Black, Other Race), education (< 9th grade, 9–11th grade, high school diploma/GED, some college/AA degree, ≥ college graduate), family income‐to‐poverty ratio (PIR), BMI, drinking status, smoking status, hypertension, and diabetes. Diabetes was defined by a physician diagnosis and/or laboratory criteria, including 2‐h plasma glucose during an oral glucose tolerance test (OGTT) ≥ 11.1 mmol/L, glycated hemoglobin (HbA1c) ≥ 6.5%, or fasting plasma glucose ≥ 7.0 mmol/L. Prediabetes was defined by self‐reported diagnosis and/or laboratory thresholds, including OGTT 2‐h glucose 7.8–11.1 mmol/L, fasting glucose 6.1–6.9 mmol/L, or HbA1c 5.7%–6.4%. Smoking status was classified as noncurrent smokers (never smokers or former smokers who had quit for > 1 year) and current smokers (currently smoking, or reporting > 1 smoking day in the past 30 days, smoking upon waking, or smoking > 2 cigarettes/day after cessation). Drinking status was defined as never drinkers (< 12 lifetime drinks) and current drinkers (≥ 12 drinks per year or > 6 drinking occasions in the past 12 months). BMI was computed as weight in kilograms divided by height in meters squared kg/m^2^.

### 2.3 Data Splitting, Machine‐Learning Feature Preprocessing and Selection

Each dataset was first divided into a training set (70%) and a locked validation set (30%) using stratified random sampling with a fixed random seed (20260830). This yielded 26 and 11 psoriasis‐comorbidity cases in the nutrient training and validation sets, respectively; 32 and 14 cases in the MPED sets; and 32 and 14 cases in the WWEIA sets. The three dietary datasets were modeled separately. The nutrient‐level dataset contained 46 dietary variables and 10 participant‐level covariates, the MPED dataset contained 37 food‐group variables and the same 10 covariates, and the WWEIA dataset contained 137 food‐item variables and the same covariates. Within each five‐fold training resampling iteration, pairwise correlation filtering (|*r*| > 0.90), shadow‐feature random‐forest selection based on the Boruta principle (Kursa and Rudnicki 2010), standardization, class‐imbalance handling, and model fitting were refitted using the analysis fold only. To retain a comparable level of model complexity across reruns, 28, 29, and 32 features were retained for the nutrient, MPED, and WWEIA representations, respectively. For class imbalance, the majority class was first moderately downsampled and the minority class was then augmented using the Synthetic Minority Over‐sampling Technique (SMOTE; Chawla et al. 2002) or the Synthetic Minority Over‐sampling Technique for Nominal and Continuous features (SMOTENC), as appropriate, within the training fold only. The locked validation set was not used for feature selection, resampling, hyperparameter tuning, threshold selection, or model selection.

### 2.4 Statistical Analyses

All statistical analyses were performed in accordance with the official NHANES analytic guidelines. Continuous variables were summarized as means and standard deviations (SD), whereas categorical variables were presented as counts and percentages. Between‐group differences were assessed using the chi‐square test or Student's *t*‐test, as appropriate.

Seven candidate models were evaluated: random forest, Light Gradient Boosting Machine (LightGBM), k‐nearest neighbors (KNN), naive Bayes, support vector machine (SVM), XGBoost, and conventional multivariable logistic regression. Hyperparameters were tuned by five‐fold stratified cross‐validation within the training set, using mean AUC‐ROC as the primary selection criterion. The candidate parameter sets and selected hyperparameters for each model are provided in Table S10. Random forest was implemented as a bagged ensemble of decision trees (Breiman 2001); LightGBM and XGBoost were implemented as gradient‐boosting tree methods (Bentéjac et al. 2021; Chen and Guestrin 2016); KNN, naive Bayes, and SVM were implemented using standard classification formulations (Cover and Hart 1967; Domingos and Pazzani 1997; Cortes and Vapnik 1995); and logistic regression served as the conventional multivariable benchmark.

Model performance was assessed using AUC‐ROC, AUC‐PR, Brier score, accuracy, sensitivity, specificity, and F1 score. Classification thresholds were determined from training out‐of‐fold predictions using Youden's index and were not selected on the validation set. Final model performance was then evaluated once in the locked validation set. For the cross‐validation‐selected model in each dietary representation, 95% confidence intervals were estimated using 1000‐sample nonparametric bootstrap resampling of the validation set. Baseline statistical analyses were conducted using IBM SPSS Statistics (v24.0) and R (v4.3.0); the revised machine‐learning analysis was performed in Python using scikit‐learn 1.8.0, xgboost 3.1.3, lightgbm 4.6.0, imbalanced‐learn 0.14.1, and shap 0.50.0. All tests were two‐sided, with *p* < 0.05 considered statistically significant where applicable.

SHAP (SHapley Additive exPlanations) was used to characterize feature contributions in the cross‐validation‐selected model for each dietary representation (Lundberg and Lee 2017; Lundberg et al. 2020). SHAP quantifies the contribution of each feature to model predictions, and mean absolute SHAP values were used to summarize global feature importance. For the logistic‐regression and XGBoost models selected in the revised analysis, SHAP results were interpreted as descriptions of fitted model behavior rather than as estimates of biological causality or intervention effects.

## 3 Results

### 3.1 Participant Characteristics by Psoriasis Comorbidity Status

Participants from three databases were included, with baseline characteristics and dietary intake differences summarized as follows:

In the NHANES database (cycles 2005–2006, 2009–2014), a total of 1016 participants were included (mean age 50.45 ± 16.05 years; 63.39% female). Among them, 37 participants had anxiety comorbid with psoriasis (mean age 53.24 ± 16.13 years). Compared to anxiety patients without psoriasis, those with comorbidity had lower intakes of dietary protein (73.44 vs. 76.29, *p* = 0.872), β‐carotene (1776.84 vs. 1903.49, *p* = 0.494), and β‐cryptoxanthin (55.93 vs. 77.36, *p* = 0.364) (Table S1).

From the MyPyramid Equivalents Database, 1215 participants were included (mean age 49.15 ± 15.59 years; 64.03% female). Comorbid anxiety and psoriasis were identified in 46 participants (mean age 50.83 ± 16.06 years). The comorbidity group showed lower intakes of citrus, melons, and berries (0.08 vs. 0.19, *p* = 0.088) and total fruits (0.86 vs. 0.89, *p* = 0.671) compared to the anxiety‐only group (Table S2).

In the WWEIA database, 1257 participants were included (mean age 49.20 ± 15.54 years; 63.64% female). Among the 46 comorbid participants (mean age 50.83 ± 16.06 years), lower intakes of fruit drinks (41.35 vs. 53.52, *p* = 0.633) and other diet drinks (16.96 vs. 18.28, *p* = 0.956) were noted relative to those with anxiety alone (Table S3).

### 3.2 Feature Selection via Machine Learning

Correlation filtering was repeated within the training workflow. In the full nutrient training set, four highly correlated variables (DR1TSFAT, DR1TMFAT, DR1TFDFE, and DR1TVK) exceeded the prespecified |*r*| > 0.90 threshold and were removed. In the MPED training set, DR1T_V_REDOR_TOTAL, DR1T_V_STARCHY_TOTAL, DR1T_G_TOTAL, and DR1T_PF_LEGUMES were removed for the same reason. No WWEIA feature exceeded the threshold in the full training set. The corresponding correlation structures are summarized in Figure 2.

**FIGURE 2:**
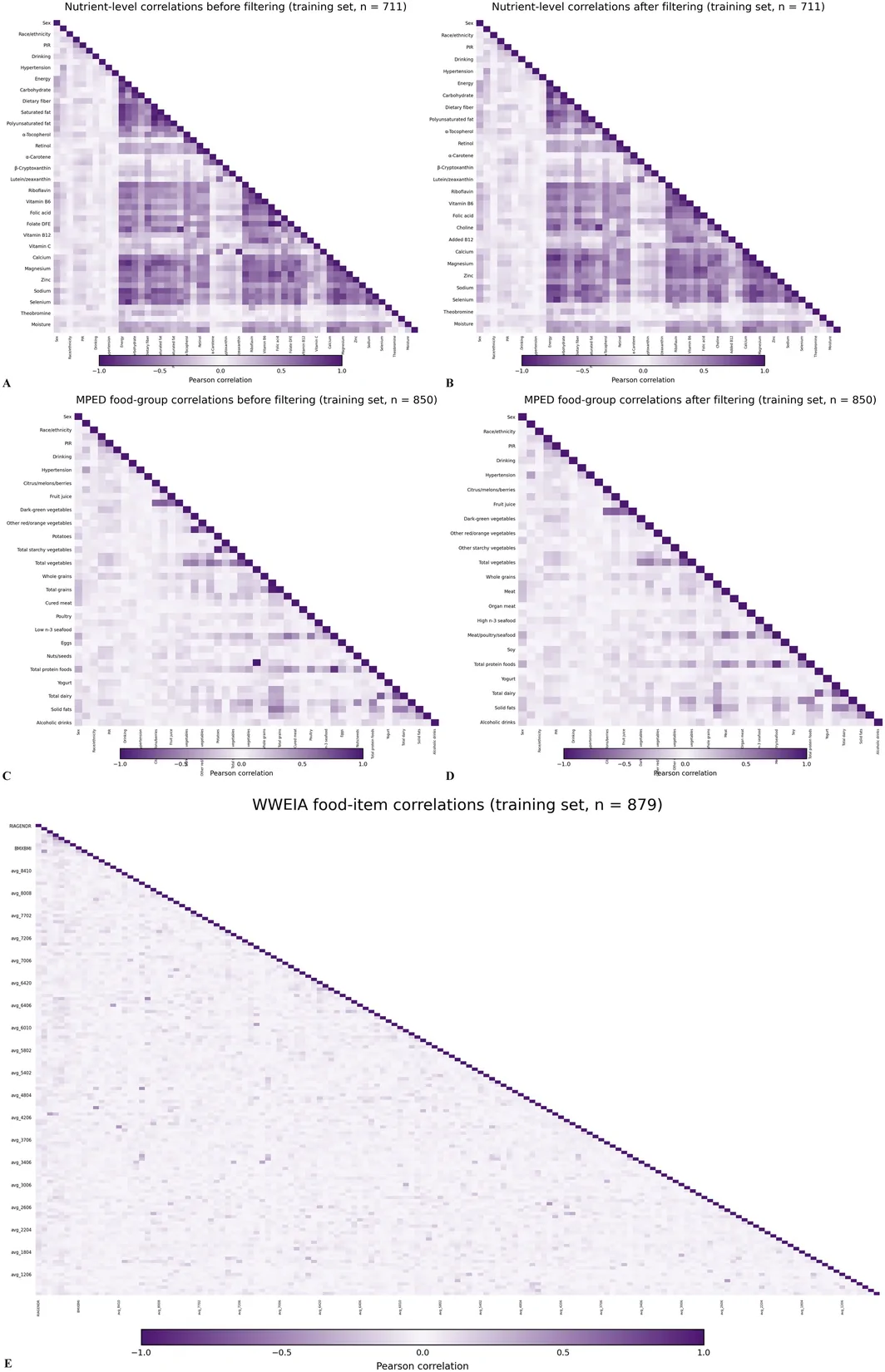
Training‐set correlation filtering of dietary features across the three dietary representations. Panels (A, B) show nutrient‐level correlation structures before and after filtering; Panels (C, D) show the corresponding MPED food‐group correlation structures, and Panel (E) shows the WWEIA food‐item correlation structure. Correlation filtering was performed within the training workflow using the prespecified threshold of |*r*| > 0.90.

Shadow‐feature random‐forest selection based on the Boruta principle retained 28 nutrient‐level features, 29 MPED food‐group features, and 32 WWEIA features in the full training datasets (Figure 3). The complete selected‐feature lists are provided in the revised Supporting Information results.

**FIGURE 3:**
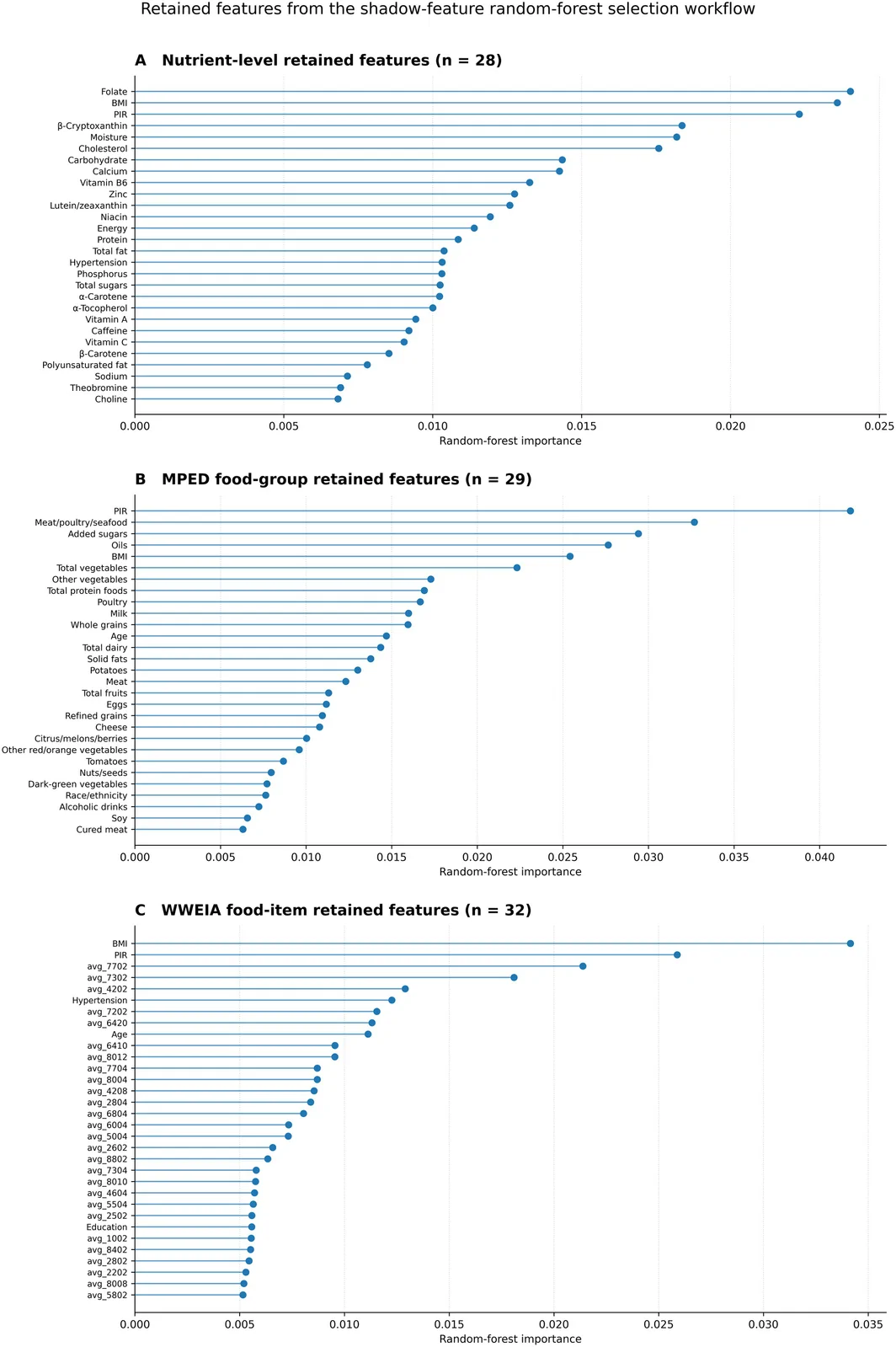
Retained features from shadow‐feature random‐forest selection across the three dietary representations. Panels (A–C) show the retained nutrient‐level, MPED food‐group, and WWEIA food‐item features, respectively. The final full‐training feature sets contained 28, 29, and 32 variables.

### 3.3 Construction and Evaluation of Machine Learning Models

Seven models—random forest, LightGBM, KNN, naive Bayes, SVM, XGBoost, and multivariable logistic regression—were evaluated separately within each dietary representation. Hyperparameter selection was based only on five‐fold stratified cross‐validation within the training set, and all final performance estimates were obtained from the locked validation sets. Overall model discrimination and performance metrics are summarized in Figures 4 and 5. Complete numeric results are provided in Tables S4–S9 and should be regarded as the definitive estimates from the leakage‐controlled analysis.

**FIGURE 4:**
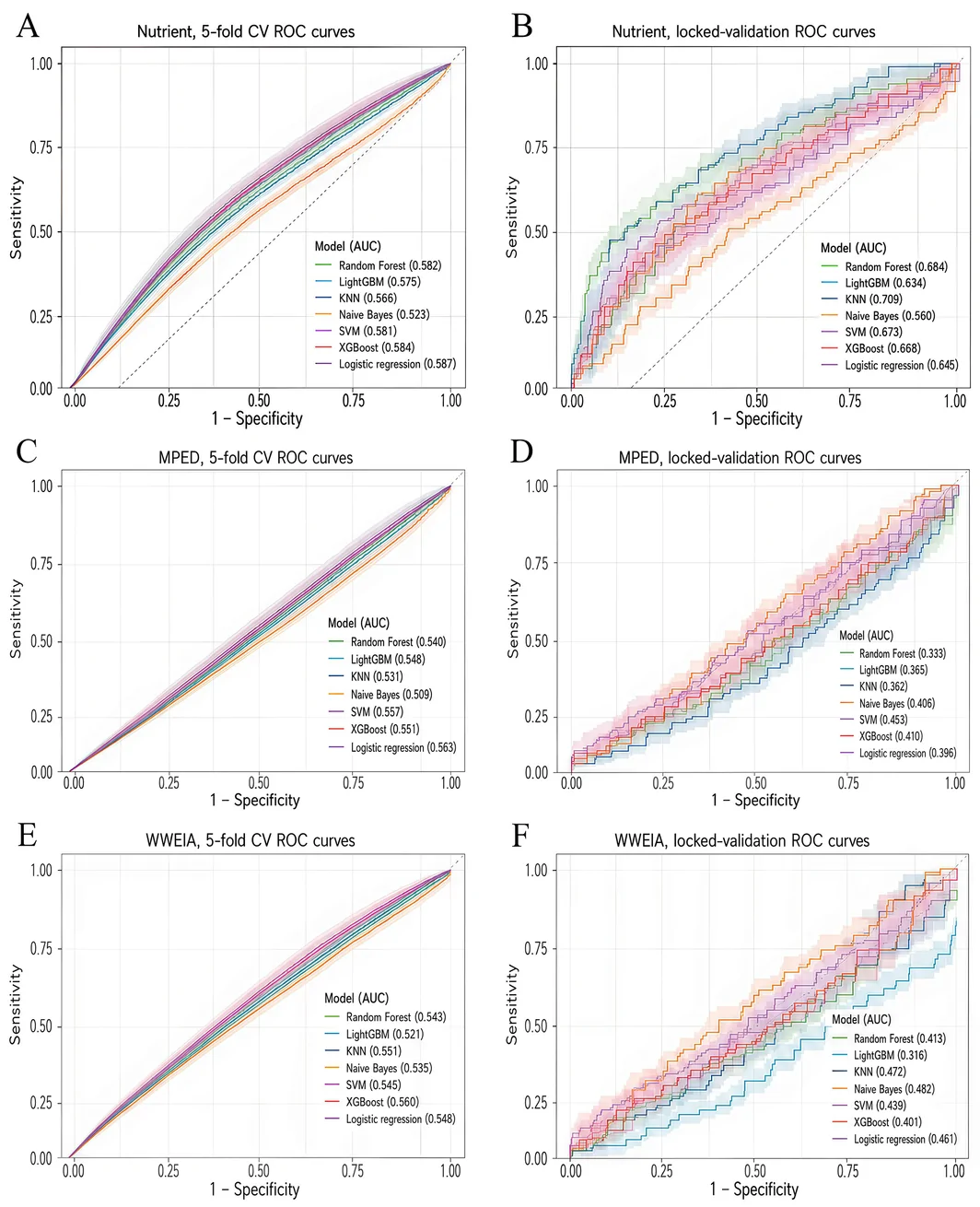
Five‐fold cross‐validation and locked‐validation receiver operating characteristic curves across the three dietary representations. Panels (A, B) show the nutrient‐level five‐fold cross‐validation and locked‐validation ROC curves, Panels (C, D) show the corresponding MPED curves, and Panels (E, F) show the WWEIA curves. Seven candidate models were compared: Random forest, LightGBM, k‐nearest neighbors, naive Bayes, support vector machine, XGBoost, and conventional multivariable logistic regression. Model selection was based on mean training cross‐validation AUC‐ROC rather than post hoc validation performance.

**FIGURE 5:**
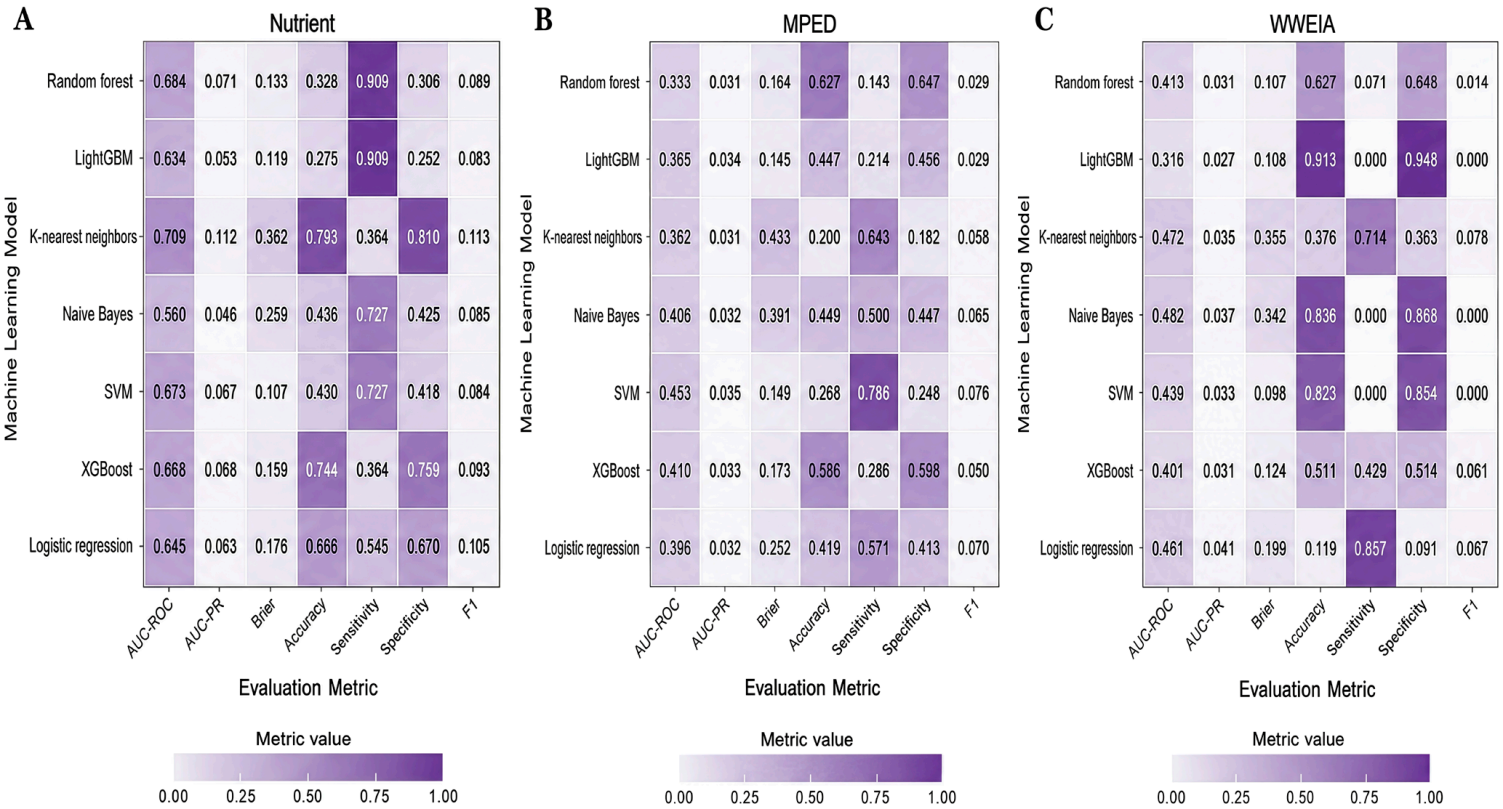
Locked‐validation performance of candidate models across the three dietary representations. Panels (A–C) show the nutrient‐level, MPED food‐group, and WWEIA food‐item representations, respectively. Model performance is summarized using AUC‐ROC, AUC‐PR, Brier score, accuracy, sensitivity, specificity, and F1 score. All metrics were calculated in the untouched locked‐validation sets using classification thresholds determined from training out‐of‐fold predictions.

For the nutrient‐level representation, multivariable logistic regression achieved the highest mean cross‐validation AUC‐ROC (0.587) and was therefore selected before validation. In the locked validation set (*n* = 305; 11 psoriasis‐comorbidity cases), it achieved an AUC‐ROC of 0.645 (95% CI 0.464–0.811), AUC‐PR of 0.063, and Brier score of 0.176. The wide confidence interval reflects the limited number of outcome events. Although several machine‐learning models showed comparable or numerically higher validation metrics, these models were not selected based on post hoc validation performance. The nutrient‐level ROC curves and model comparisons are shown in Figures 4A,B and 5A.

For the MPED food‐group representation, logistic regression had the highest mean cross‐validation AUC‐ROC (0.563). In the locked validation set (*n* = 365; 14 cases), the model yielded an AUC‐ROC of 0.396 (95% CI 0.256–0.539), AUC‐PR of 0.032, and Brier score of 0.252. These results indicate limited and unstable held‐out discrimination in the present sample. However, the model‐derived features may still provide exploratory information for subsequent hypothesis generation and external validation. The MPED ROC curves and performance metrics are presented in Figures 4C,D and 5B.

For the WWEIA food‐item representation, XGBoost was selected from training cross‐validation (mean AUC‐ROC 0.560). In the locked validation set (*n* = 378; 14 cases), the model achieved an AUC‐ROC of 0.401 (95% CI 0.273–0.537), AUC‐PR of 0.031, and Brier score of 0.124. These findings indicate limited held‐out discrimination and suggest that greater dietary granularity did not improve out‐of‐sample performance in this low‐event dataset. Nevertheless, the model may assist in prioritizing candidate food‐item features for future investigation. The WWEIA ROC curves and performance metrics are shown in Figures 4E,F and 5C.

Across all dietary representations, the leakage‐controlled rerun did not reproduce the high validation AUCs observed in the previous analysis. Logistic regression was selected for two of the three dietary representations, and no consistent performance advantage of machine learning over conventional multivariable modeling was observed. Nevertheless, machine learning and conventional regression provided complementary perspectives across dietary representations, with machine learning retaining value for high‐dimensional feature exploration and prioritization.

### 3.4 Model Interpretation Using SHAP

SHAP (SHapley Additive exPlanations) was used to characterize feature contributions in the cross‐validation‐selected model for each dietary representation. Positive SHAP values indicate contributions toward a higher model‐predicted probability of psoriasis comorbidity, whereas negative SHAP values indicate contributions toward a lower predicted probability. Global SHAP summaries across the three dietary representations are presented in Figure 6.

**FIGURE 6:**
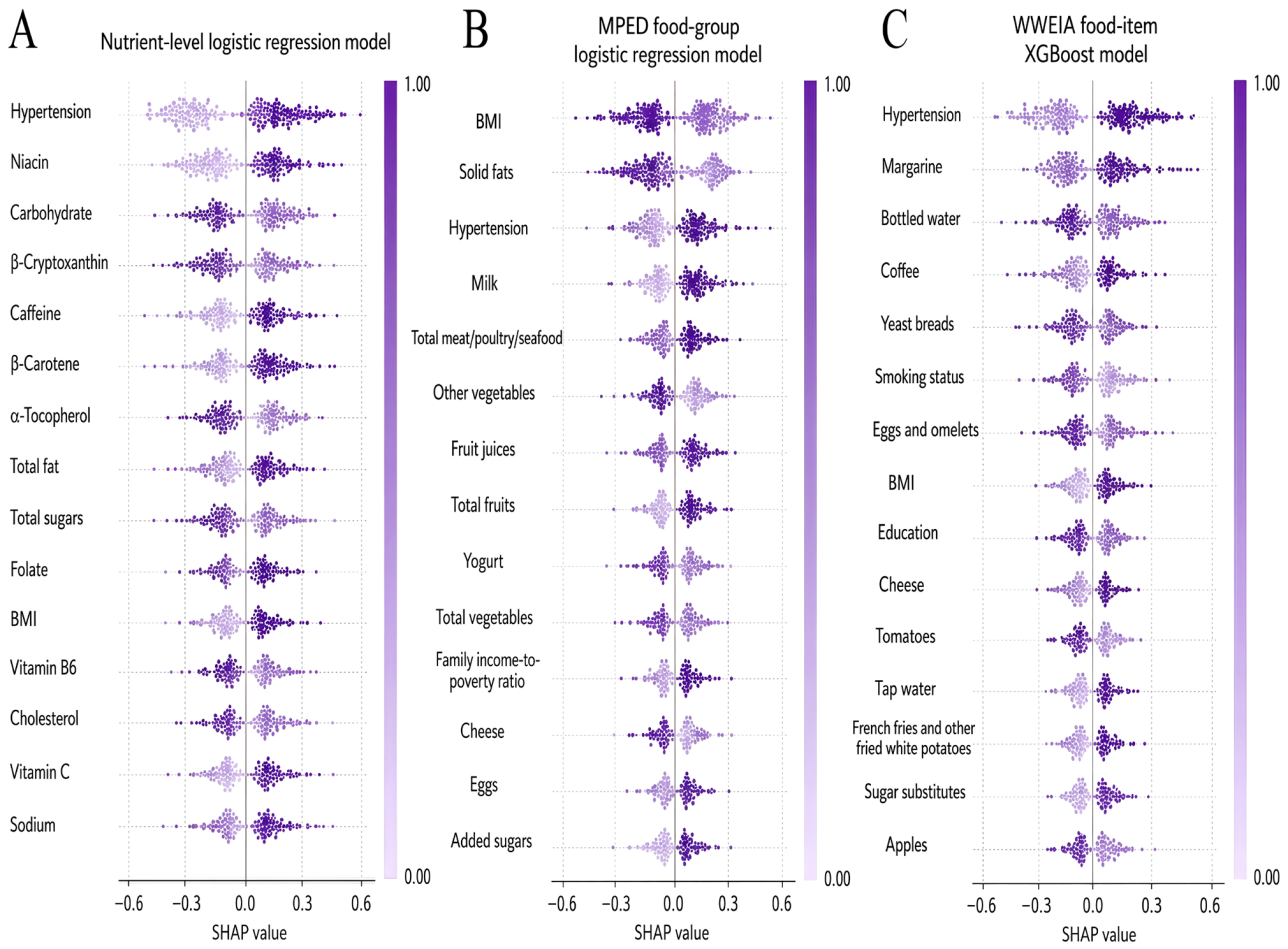
Global SHAP interpretation of the cross‐validation‐selected models across the three dietary representations. Panels (A–C) show global SHAP summaries for the nutrient‐level logistic regression model, MPED food‐group logistic regression model, and WWEIA XGBoost model, respectively. Positive SHAP values indicate contributions toward a higher model‐predicted probability of psoriasis comorbidity, whereas negative SHAP values indicate contributions toward a lower predicted probability. Feature colors represent relative feature values. SHAP results are interpreted as model‐derived signals rather than causal effects.

In the nutrient‐level logistic regression model, niacin showed the largest dietary SHAP contribution, with higher values contributing toward a lower model‐predicted probability of psoriasis comorbidity. Carbohydrate intake, β‐cryptoxanthin, caffeine, and β‐carotene showed similar contributions, whereas α‐tocopherol, total fat, total sugars, folate, and other nutrient variables contributed in the opposite direction. Hypertension was the strongest non‐dietary contributor (Figure 6A).

In the MPED logistic regression model, the leading SHAP contributions involved family income‐to‐poverty ratio (PIR), added sugars, BMI, alcoholic drinks, total vegetables, eggs, and other food‐group variables (Figure 6B). In the WWEIA XGBoost model, margarine, hypertension, tap water, mayonnaise, BMI, coffee, selected grain categories, and soft drinks ranked among the leading contributors (Figure 6C).

Given the limited held‐out discrimination of the MPED and WWEIA models, SHAP‐derived rankings should be interpreted as exploratory model‐behavior signals rather than biological effects or causal dietary associations. These features may help prioritize candidates for future validation in larger prospective datasets.

## 4 Discussion

### 4.1 Multi‐Granularity Dietary Modeling and Explainable Machine Learning for Risk Stratification

Using the same outcome and evaluation framework, we compared nutrient‐, food‐group‐, and food‐item‐level dietary representations to test whether increasing dietary granularity improved the identification of psoriasis comorbidity among adults with anxiety. Recent NHANES‐based studies have combined dietary variables with machine learning and explainability (Qi et al. 2025), while anxiety–psoriasis research has largely relied on conventional association analyses (Bai and Ma 2025). The present study adds a direct multi‐representation comparison under a locked‐validation framework. Importantly, however, the revised analysis shows that greater dietary granularity did not automatically yield better out‐of‐sample performance, highlighting the importance of validating apparent gains from high‐dimensional dietary modeling rather than inferring added value from training performance alone.

The comparison with conventional multivariable logistic regression also clarifies the role of machine learning in this dataset. Although tree‐based and kernel models can capture nonlinear relationships and interactions, logistic regression achieved the highest cross‐validation AUC‐ROC for both the nutrient and MPED representations, whereas XGBoost was selected for WWEIA. Machine learning and conventional regression therefore provided complementary perspectives across dietary representations, with machine learning retaining value for high‐dimensional feature exploration and prioritization. However, the limited number of outcome events and the weak held‐out discrimination of the MPED and WWEIA models indicate that model complexity could not compensate for limited independent outcome information, and no consistent predictive superiority of machine learning over conventional multivariable modeling was demonstrated.

Among the three representations, the nutrient‐level model showed the strongest held‐out signal (AUC‐ROC 0.645), although the estimate remained imprecise and its confidence interval included 0.5. The MPED and WWEIA models showed limited and unstable held‐out discrimination (AUC‐ROC 0.396 and 0.401, respectively), but they still identified complementary food‐group‐ and food‐item‐level features that can be prioritized in future larger‐scale analyses. Accordingly, these representations should not yet be regarded as clinically predictive, while nutrient‐level dietary information appears to be the most promising starting point for subsequent model refinement.

SHAP analysis remained useful for identifying which variables drove the fitted models, but the revised results also illustrate why predictive importance and biological importance must be separated. In the nutrient model, niacin and several other dietary variables contributed materially to predictions, while hypertension and other participant characteristics were also influential. For the food‐group and food‐item models, feature rankings arose from models with weak held‐out discrimination and therefore provide hypotheses for future study rather than evidence of robust dietary risk factors.

### 4.2 Nutrient‐Level Predictive Signals and Model Interpretation

Within the nutrient‐level model, niacin was the most influential dietary feature and higher intake contributed toward a lower model‐predicted probability of psoriasis comorbidity. β‐cryptoxanthin, carbohydrate intake, caffeine, and β‐carotene showed similar negative model contributions, whereas α‐tocopherol, total fat, total sugars, and folate contributed toward higher predicted probability. These directions differ from the earlier analysis and reinforce that feature effects are sensitive to model specification and validation strategy in a low‐event setting.

The niacin signal remains biologically plausible because niacin contributes to NAD+ metabolism and sirtuin‐mediated regulation of cellular energy and inflammatory signaling (Cantó et al. 2015; Covarrubias et al. 2021). Carotenoid‐related signals may also be relevant to redox balance and immune homeostasis. However, the present findings do not support translating individual nutrient SHAP values into supplementation targets. Their main value is to identify candidate nutritional pathways that can be tested in larger prospective datasets and intervention studies.

The nutrient model's modest discrimination also suggests a practical direction for subsequent research. Rather than increasing feature granularity alone, future work may benefit more from repeated dietary assessment, larger numbers of psoriasis outcomes, and integration of clinical variables that capture inflammatory burden and treatment status. Such data could clarify whether the nutrient‐level signals observed here are reproducible and clinically actionable.

### 4.3 Food‐Group and Food‐Item Representations

The food‐group and food‐item representations showed limited and unstable held‐out discrimination. Nevertheless, variables such as margarine, added sugars, vegetables, coffee, and soft drinks ranked among the leading model contributors, providing a focused set of candidate dietary features for further evaluation in larger prospective studies. Given the corresponding MPED and WWEIA validation AUC‐ROCs of 0.396 and 0.401, these features should not be labeled as protective or harmful dietary factors on the basis of the present models.

This does not negate the broader epidemiological evidence linking dietary quality, ultra‐processed food consumption, and psoriasis‐related inflammation (Peng et al. 2025; Perez‐Bootello et al. 2025; Sivasami et al. 2023; Kashani et al. 2021; Millsop et al. 2014; Biełach‐Bazyluk et al. 2025; Park et al. 2017; Sonomoto et al. 2023; Herbert et al. 2018). Instead, it indicates that the present anxiety–psoriasis sample is too sparse to establish a stable multivariable food‐pattern classifier. The contrast between biologically plausible dietary literature and weak locked‐validation performance is clinically informative: mechanistic plausibility alone is insufficient for individual‐level prediction, and predictive claims require independent validation.

Coffee illustrates this distinction. Coffee appeared among the influential WWEIA features, but its direction within the fitted model should not be generalized because the model itself did not discriminate the outcome reliably. Existing evidence on coffee, inflammation, anxiety, and psoriasis remains heterogeneous (Min et al. 2023; Barrea et al. 2018; Festugato 2011; Song et al. 2024; Preedalikit et al. 2024; Liu et al. 2024), suggesting that future studies should consider dose, co‐exposures, sleep, anxiety severity, and other potential effect modifiers when evaluating coffee‐related associations.

Accordingly, the food‐level results are best viewed as a prioritization map for subsequent nutritional epidemiology rather than as a basis for dietary prescriptions. Future studies should test these signals in larger samples with repeated dietary measurements and sufficient numbers of incident psoriasis outcomes.

### 4.4 Clinical and Public Health Implications

The current models are not ready for screening or treatment decisions. Nevertheless, the nutrient‐level representation showed the strongest validation signal and may provide a practical starting point for future nutrition‐informed model refinement. In this setting, machine‐learning methods may complement conventional regression by screening and prioritizing high‐dimensional dietary features rather than by providing evidence of superior clinical prediction. Integration with inflammatory markers, treatment information, and repeated clinical assessments may further improve model development in psychodermatologic populations.

From a public‐health perspective, the identified nutrient‐, food‐group‐, and food‐item‐level features provide a focused set of modifiable exposures for prospective investigation, but intervention targets should be derived from prospective and interventional evidence rather than from the present prediction models alone. Larger event‐rich cohorts, repeated dietary measurements, and external validation will be necessary to determine whether these signals can ultimately support individualized dietary assessment or coordinated dermatologic–psychiatric management.

### 4.5 Limitations and Future Directions

Several limitations should be considered. Anxiety was identified from prescription‐medication indications, which may miss untreated individuals and do not necessarily correspond to a formally confirmed psychiatric diagnosis. Psoriasis status was self‐reported and may also be subject to misclassification. Dietary exposure was derived from 24‐h recall data and is therefore subject to recall and reporting error. The cross‐sectional design does not establish temporal ordering. Most importantly, the number of psoriasis‐comorbidity events was small: the locked validation sets contained only 11 nutrient‐level cases and 14 cases each in the MPED and WWEIA datasets. SMOTE/SMOTENC improves representation of the minority class during training but does not create independent outcome information, and in a small‐event setting it may amplify unstable decision boundaries. The resulting confidence intervals were wide, and the lack of external validation further limits transportability. Larger prospective cohorts with repeated dietary assessment and independent validation are needed before clinical use can be considered.

## 5 Conclusion

In this multi‐representation analysis, the leakage‐controlled rerun yielded distinct but modest predictive signals across dietary representations. The nutrient‐level logistic regression model showed the strongest held‐out discrimination (AUC‐ROC 0.645), whereas the MPED and WWEIA models showed limited and unstable validation performance. Conventional logistic regression was selected for two of the three dietary representations, and no consistent performance advantage of machine learning over conventional multivariable modeling was observed. Nevertheless, machine learning and conventional regression provided complementary perspectives across dietary representations, with machine learning retaining value for high‐dimensional feature exploration and prioritization. SHAP analysis further prioritized niacin, carbohydrate intake, β‐cryptoxanthin, fat‐related variables, hypertension, BMI, and selected food categories as candidate features for subsequent validation.

The current models are not ready for immediate risk prediction or treatment decisions. Their main value lies in prioritizing dietary and clinical variables for larger prospective studies, with nutrient‐level information providing the most promising starting point for further model refinement. Larger event‐rich cohorts, repeated dietary assessment, and independent external validation will be necessary to determine whether nutrition‐informed assessment can ultimately contribute to earlier recognition and more individualized management of psoriasis comorbidity among people with anxiety.

## Author Contributions

**Canni Gao:** writing – original draft. **Xinxin Duan:** methodology. **Mei Luan:** data curation. **Weijie Huang:** validation. **Guangdong Wang:** visualization. **Zhihong Xu:** writing – original draft, writing – review and editing.

## Funding

The author(s) declare financial support was received for the research, authorship, and/or publication of this article. This work was supported by grants from the Xiamen Medical Association Clinical Research Support Special Fund Project (project no. HY202501015), and the APC payment was supported by the corresponding author, Prof. Zhihong Xu.

## Ethics Statement

This study was conducted in compliance with the Declaration of Helsinki. The data analyzed in this study were obtained from three publicly available, de‐identified databases (NHANES, MPED, and WWEIA), which have been approved by their respective institutional review boards (IRBs) for unrestricted public use in secondary research. Since all data are anonymized and do not contain any personal identifiable information, ethical approval and informed consent from individual participants were waived for this study, in accordance with the relevant guidelines for secondary analysis of publicly accessible health data.

## Consent

All authors have approved the final version of this manuscript for submission and publication. The data used in this study are derived from publicly available, de‐identified databases (NHANES, MPED, and WWEIA), which do not require additional consent for publication of results based on secondary analysis. No identifiable private information is included in this manuscript, and all authors confirm that the work described has not been published previously and is not under consideration for publication elsewhere.

## Conflicts of Interest

The authors declare no conflicts of interest.

## Supporting information


**Table S1:** Baseline Characteristics and Dietary Micronutrient Intake by Psoriasis Status—NHANES (2005–2006, 2009–2014) Note: Data are presented as mean ± SD or *n* (%). *p*‐Values were derived from Student's *t*‐test for continuous variables and chi‐square test for categorical variables. SD, standard deviation.
**Table S2:** Food Group Intake by Psoriasis Status—MyPyramid Equivalents Database Note: Intake levels are expressed as mean ± SD. Statistical comparisons were performed using Student's *t*‐test. SD, standard deviation.
**Table S3:** Specific Food and Beverage Intake by Psoriasis Status—WWEIA Database Note: Values represent mean daily intake (g) ± SD. *p*‐Values were calculated using Student's *t*‐test. SD, standard deviation.
**Table S4:** Best tuned five‐fold cross‐validation performance for the Nutrient representation.
**Table S5:** Locked‐validation performance for the Nutrient representation.
**Table S6:** Best tuned five‐fold cross‐validation performance for the MPED representation.
**Table S7:** Locked‐validation performance for the MPED representation.
**Table S8:** Best tuned five‐fold cross‐validation performance for the WWEIA representation.
**Table S9:** Locked‐validation performance for the WWEIA representation.
**Table S10:** Hyperparameter search space and selected settings.

## Data Availability

The data used in this study were obtained from three public open databases, namely NHANES, MPED, and WWEIA. All relevant data are publicly accessible in accordance with the respective database policies, and no additional restrictions apply to data access: NHANES official website (https://www.cdc.gov/nchs/nhanes/index.html); NHANES questionnaires, datasets, and documentation (https://wwwn.cdc.gov/nchs/nhanes/); NHANES dietary data/What We Eat in America (https://wwwn.cdc.gov/Nchs/Nhanes/Products/WhatWeEat.aspx); WWEIA data tables (https://www.ars.usda.gov/northeast‐area/beltsville‐md‐bhnrc/beltsville‐human‐nutrition‐research‐center/food‐surveys‐research‐group/docs/wweia‐data‐tables/); MPED databases for downloading (https://www.ars.usda.gov/northeast‐area/beltsville‐md‐bhnrc/beltsville‐human‐nutrition‐research‐center/food‐surveys‐research‐group/docs/mped‐databases‐for‐downloading/).
