# Supplementary material for: Nutrient‐, Food‐Group‐, and Food‐Item‐Level Dietary Signatures of Psoriasis Comorbidity Among Adults With Anxiety Disorder: An Explainable Machine Learning Study
Source: Food Sci Nutr. 2026 Oct 21;14(10):e72482. doi: 10.1002/fsn3.72482 (PMC13685531; doi:10.1002/fsn3.72482)
Supplement: Supplementary file 1 [file FSN3-14-e72482-s001.docx]

***Supplementary Material***

| **Characteristic** | **N** | **Overall** | **No Psoriasis** | **Psoriasis** | **p-value** |
| --- | --- | --- | --- | --- | --- |
|  |  | **N = 1,016** | **N = 979** | **N = 37** |  |
| Sex b, n(%) | 1,016 |  |  |  | 0.875 |
| FeMale |  | 644 (63.39%) | 621 (63.43%) | 23 (62. 16%) |  |
| Male |  | 372 (36.61%) | 358 (36.57%) | 14 (37.84%) |  |
| Age a, Mean ± SD | 1,016 | 50.45 ± 16.05 | 50.34 ± 16.04 | 53.24 ± 16.13 | 0.290 |
| Race/ethnicity b, n(%) | 1,016 |  |  |  | 0.339 |
| Mexican |  | 76 (7.48%) | 71 (7.25%) | 5 (13.51%) |  |
| Other Hispanic |  | 62 (6. 10%) | 60 (6. 13%) | 2 (5.41%) |  |
| Non-Hispanic White |  | 676 (66.54%) | 653 (66.70%) | 23 (62. 16%) |  |
| Non-Hispanic Black |  | 138 (13.58%) | 135 (13.79%) | 3 (8. 11%) |  |
| Other Race |  | 64 (6.30%) | 60 (6. 13%) | 4 (10.81%) |  |
| Education b, n(%) | 1,016 |  |  |  | 0.213 |
| <9th grade |  | 54 (5.31%) | 51 (5.21%) | 3 (8. 11%) |  |
| 9–11th grade |  | 133 (13.09%) | 129 (13. 18%) | 4 (10.81%) |  |
| High school diploma/GED |  | 236 (23.23%) | 222 (22.68%) | 14 (37.84%) |  |
| Some College/AA degree |  | 337 (33. 17%) | 328 (33.50%) | 9 (24.32%) |  |
| ≥College graduate |  | 256 (25.20%) | 249 (25.43%) | 7 (18.92%) |  |
| [Family.income.to.poverty.ratio a](Family.income.to.poverty.ratioa), Mean ± SD | 1,016 | 2.55 ± 1.70 | 2.56 ± 1.70 | 2.15 ± 1.84 | 0.072 |
| Energy a, Mean ± SD | 1,016 | 2,000.95 ± 837.05 | 1,997.54 ± 833.82 | 2,091.22 ± 926.50 | 0.487 |
| protein a, Mean ± SD | 1,016 | 76.18 ± 34.49 | 76.29 ± 34.67 | 73.44 ± 29.80 | 0.872 |
| Carbohydrate a, Mean ± SD | 1,016 | 245.19 ± 112.23 | 244.86 ± 111.61 | 253.98 ± 128.92 | 0.639 |
| Total.Sugar a, Mean ± SD | 1,016 | 112.78 ± 72.10 | 112.34 ± 71.42 | 124.27 ± 88.59 | 0.680 |
| Dietary.fiber a, Mean ± SD | 1,016 | 15.90 ± 8.80 | 15.87 ± 8.62 | 16.62 ± 12.82 | 0.627 |

| **Characteristic** | **N** | **Overall**  **N = 1,016** | **No Psoriasis**  **N = 979** | **Psoriasis**  **N = 37** | **p-value** |
| --- | --- | --- | --- | --- | --- |
| Total.Fat a, Mean ± SD | 1,016 | 75.53 ± 38.59 | 75.37 ± 38.64 | 79.78 ± 37.47 | 0.318 |
| Saturated.fatty.acids a, Mean ± SD | 1,016 | 24.97 ± 13.91 | 24.92 ± 13.96 | 26.35 ± 12.42 | 0.228 |
| Monounsaturated.fatty.acids a, Mean ± SD | 1,016 | 26.66 ± 14.44 | 26.60 ± 14.46 | 28.09 ± 13.92 | 0.354 |
| Polyunsaturated.fatty.acids a, Mean ± SD | 1,016 | 17.16 ± 10.12 | 17.12 ± 10.10 | 18.35 ± 10.79 | 0.457 |
| Cholesterol a, Mean ± SD | 1,016 | 259.24 ± 173.09 | 258.95 ± 173.86 | 266.69 ± 153.47 | 0.420 |
| Vitamin.E a, Mean ± SD | 1,016 | 8.25 ± 6.51 | 8.19 ± 6.02 | 10.07 ± 14.36 | 0.957 |
| alpha.tocopherol a, Mean ± SD | 1,016 | 1.06 ± 4.30 | 1.00 ± 3.80 | 2.82 ± 11.17 | 0.340 |
| Retinol a, Mean ± SD | 1,016 | 437.78 ± 420.55 | 436.19 ± 421.11 | 479.82 ± 408.58 | 0.562 |
| Vitamin.A a, Mean ± SD | 1,016 | 613.16 ± 487.66 | 611.88 ± 487.85 | 646.91 ± 488.01 | 0.822 |
| Alpha-carotene a, Mean ± SD otene | 1,016 | 345.46 ± 673.19 | 343.13 ± 656.23 | 407.23 ±  1,036.45 | 0.485 |
| Beta.carotene a, Mean ± SD | 1,016 | 1,898.87 ± 2,519.75 | 1,903.49 ± 2,516.62 | 1,776.84 ± 2,633.98 | 0.494 |
| Beta.cryptoxanthin a, Mean ± SD | 1,016 | 76.58 ± 197.51 | 77.36 ± 200.57 | 55.93 ± 80.59 | 0.364 |
| Lycopene a, Mean ± SD | 1,016 | 4,749.83 ± 7,388.04 | 4,769.00 ± 7,447.52 | 4,242.53 ± 5,643.80 | 0.662 |
| Lutein.zeaxanthin a, Mean ± SD | 1,016 | 1,411.07 ± 2,319.11 | 1,410.93 ± 2,316.04 | 1,414.85 ± 2,431.79 | 0.770 |
| Vitamin.B1 a, Mean ± SD | 1,016 | 1.55 ± 0.82 | 1.55 ± 0.79 | 1.56 ± 1.37 | 0.333 |
| Vitamin.B2 a, Mean ± SD | 1,016 | 2.15 ± 1.31 | 2.14 ± 1.25 | 2.32 ± 2.34 | 0.945 |
| Niacin a, Mean ± SD | 1,016 | 24.42 ± 13.79 | 24.43 ± 13.17 | 24.06 ± 25.55 | 0.089 |
| Vitamin.B6 a, Mean ± SD | 1,016 | 2.07 ± 1.48 | 2.07 ± 1.41 | 2.05 ± 2.80 | 0.014 |
| Total.folate a, Mean ± SD | 1,016 | 393.54 ± 246 05 | 391.75 ± 233 31 | 440.97 ± 475 07 | 0.616 |

| **Characteristic** | **N** | **Overall**  **N = 1,016** | **No Psoriasis**  **N = 979** | **Psoriasis**  **N = 37** | **p-value** |
| --- | --- | --- | --- | --- | --- |
| Folic.acid a, Mean ± SD | 1,016 | 190.57 ± 189.62 | 188.36 ± 177.05 | 248.93 ± 398.12 | 0.847 |
| Food.folate a, Mean ± SD | 1,016 | 203.03 ± 115.29 | 203.45 ± 115.72 | 192.03 ± 104.03 | 0.559 |
| Folate.DFE. a, Mean ± SD | 1,016 | 527.02 ± 369.36 | 523.68 ± 347.11 | 615.30 ± 751.59 | 0.697 |
| Total.choline a, Mean ± SD | 1,016 | 306.13 ± 154.30 | 306.73 ± 155.11 | 290.34 ± 131.87 | 0.751 |
| Vitamin.B12 a, Mean ± SD | 1,016 | 5.33 ± 5.99 | 5.32 ± 5.91 | 5.51 ± 8.01 | 0.307 |
| Added.vitamin.B12 a, Mean ± SD | 1,016 | 1.26 ± 2.79 | 1.25 ± 2.68 | 1.57 ± 5.00 | 0.198 |
| Vitamin.C a, Mean ± SD | 1,016 | 77.26 ± 80.82 | 77.08 ± 79.32 | 82.04 ± 115.08 | 0.941 |
| Vitamin.K a, Mean ± SD | 1,016 | 103.38 ± 114.32 | 103.48 ± 114.69 | 100.67 ± 105.57 | 0.983 |
| Calcium a, Mean ± SD | 1,016 | 965.16 ± 585.59 | 961.01 ± 582.52 | 1,074.82 ± 661.25 | 0.259 |
| Phosphorus a, Mean ± SD | 1,016 | 1,317.69 ± 593.14 | 1,315.36 ± 592.02 | 1,379.14 ± 627.43 | 0.424 |
| Magnesium a, Mean ± SD | 1,016 | 286.26 ± 133.53 | 286.07 ± 132.23 | 291.23 ± 166.38 | 0.654 |
| Iron a, Mean ± SD | 1,016 | 14.78 ± 8.72 | 14.72 ± 8.21 | 16.17 ± 17.71 | 0.522 |
| Zinc a, Mean ± SD | 1,016 | 10.91 ± 6.35 | 10.85 ± 5.95 | 12.39 ± 13.12 | 0.831 |
| Copper a, Mean ± SD | 1,016 | 1.20 ± 0.78 | 1.20 ± 0.78 | 1.20 ± 0.69 | 0.611 |
| Sodium a, Mean ± SD | 1,016 | 3,269.70 ± 1,493.89 | 3,266.82 ± 1,494.38 | 3,346.01 ± 1,499.28 | 0.709 |
| Potassium a, Mean ± SD | 1,016 | 2,543.98 ± 1,098.54 | 2,547.00 ± 1,098.23 | 2,464.19 ± 1, 118.78 | 0.661 |
| Selenium a, Mean ± SD | 1,016 | 104.70 ± 51.29 | 104.75 ± 51.28 | 103.41 ± 52.30 | 0.948 |
| Caffeine a, Mean ± SD | 1,016 | 181.36 ± 191.85 | 181.37 ± 192.97 | 181.03 ± 161.48 | 0.514 |
| Theobromine a, Mean ± SD | 1,016 | 38 05 ± 56 99 | 38 07 ± 57 06 | 37 45 ± 55 86 | 0 997 |

| **Characteristic** | **N** | **Overall**  **N = 1,016** | **No Psoriasis**  **N = 979** | **Psoriasis**  **N = 37** | **p-value** |
| --- | --- | --- | --- | --- | --- |
| Alcohol a, Mean ± SD | 1,016 | 9.06 ± 21.77 | 8.88 ± 20.60 | 13.72 ± 42.46 | 0.260 |
| Moisture a, Mean ± SD | 1,016 | 2,901.71 ± 1,392.69 | 2,892.93 ± 1,387.38 | 3, 133.91 ± 1,528.81 | 0.230 |
| Vitamin.D a, Mean ± SD | 1,016 | 4.71 ± 4.54 | 4.70 ± 4.50 | 4.98 ± 5.63 | 0.666 |
| Smoking.status b, n(%) | 1,016 |  |  |  | 0.705 |
| No |  | 713 (70. 18%) | 686 (70.07%) | 27 (72.97%) |  |
| Yes |  | 303 (29.82%) | 293 (29.93%) | 10 (27.03%) |  |
| Drinking.status b, n(%) | 1,016 |  |  |  | 0.784 |
| No |  | 103 (10. 14%) | 99 (10. 11%) | 4 (10.81%) |  |
| Yes |  | 913 (89.86%) | 880 (89.89%) | 33 (89. 19%) |  |
| BMI a, Mean ± SD | 1,016 | 29.82 ± 7.29 | 29.68 ± 7.20 | 33.48 ± 8.56 | 0.006 |
| Hypertension b, n(%) | 1,016 |  |  |  | <0.001 |
| No |  | 581 (57. 19%) | 570 (58.22%) | 11 (29.73%) |  |
| Yes |  | 435 (42.81%) | 409 (41.78%) | 26 (70.27%) |  |
| Diabetes b, n(%) | 1,016 |  |  |  | 0.029 |
| No |  | 835 (82. 19%) | 807 (82.43%) | 28 (75.68%) |  |
| Yes |  | 121 (11.91%) | 112 (11.44%) | 9 (24.32%) |  |
| Borderline |  | 60 (5.91%) | 60 (6. 13%) | 0 (0.00%) |  |

a: Student t-test, b: Chi-square test, SD: standard deviation

Supplementary Table 1. Baseline Characteristics and Dietary Micronutrient Intake by Psoriasis Status – NHANES (2005–2006, 2009–2014) Note: Data are presented as mean ± SD or n (%). p-values were derived from Student’s t-test for continuous variables and chi-square test for categorical variables. Abbreviations: SD, standard deviation.

| **Characteristic** | **N** | **Overall**  **N = 1,215** | **No Psoriasis**  **N = 1,169** | **Psoriasis**  **N = 46** | **p-value** |
| --- | --- | --- | --- | --- | --- |
| Sex | 1,215 |  |  |  | 0.887 |
| FeMale |  | 778 (64.03%) 749 (64.07%) | | 29 (63.04%) |  |
| Male |  | 437 (35.97%) 420 (35.93%) | | 17 (36.96%) |  |
| Age a, Mean ± SD | 1,215 | 49.15 ± 15.59 49.08 ± 15.58 | | 50.83 ± 16.06 | 0.482 |
| Race/ethnicity | 1,215 |  |  |  | 0.218 |
| Mexican |  | 96 (7.90%) 90 (7.70%) | | 6 (13.04%) |  |
| Other Hispanic |  | 71 (5.84%) 68 (5.82%) | | 3 (6.52%) |  |
| Non-Hispanic White |  | 822 (67.65%) 793 (67.84%) | | 29 (63.04%) |  |
| Non-Hispanic Black |  | 154 (12.67%) 151 (12.92%) | | 3 (6.52%) |  |
| Other Race |  | 72 (5.93%) 67 (5.73%) | | 5 (10.87%) |  |
| Education | 1,215 |  |  |  | 0.551 |
| <9th grade |  | 63 (5. 19%) 60 (5. 13%) | | 3 (6.52%) |  |
| 9–11th grade |  | 152 (12.51%) 146 (12.49%) | | 6 (13.04%) |  |
| High school diploma/GED |  | 288 (23.70%) 273 (23.35%) | | 15 (32.61%) |  |
| Some College/AA degree |  | 411 (33.83%) 399 (34. 13%) | | 12 (26.09%) |  |
| ≥College graduate |  | 301 (24.77%) 291 (24.89%) | | 10 (21.74%) |  |
| [Family.income.to.poverty.ratio a](Family.income.to.poverty.ratioa), Mean ± SD | 1,215 | 2.64 ± 1.72 2.65 ± 1.71 | | 2.18 ± 1.82 | 0.044 |
| Smoking.status | 1,215 |  |  |  | 0.813 |
| No |  | 812 (66.83%) 782 (66.89%) | | 30 (65.22%) |  |
| Yes |  | 403 (33. 17%) 387 (33. 11%) | | 16 (34.78%) |  |
| Drinking.status | 1,215 |  |  |  | >0.999 |
| No |  | 117 (9.63%) | 113 (9.67%) | 4 (8.70%) |  |
| Yes |  | 1,098 (90.37%) | 1,056 (90.33%) | 42 (91.30%) |  |
| BMI a, Mean ± SD | 1,215 | 29.68 ± 7.17 | 29.55 ± 7.10 | 32.87 ± 8.18 | 0.005 |

| **Characteristic** | **N** | **Overall**  **N = 1,215** | **No Psoriasis**  **N = 1,169** | **Psoriasis**  **N = 46** | **p-value** |
| --- | --- | --- | --- | --- | --- |
| Hypertension | 1,215 |  |  |  | 0.005 |
| No |  | 720 (59.26%) | 702 (60.05%) | 18 (39. 13%) |  |
| Yes |  | 495 (40.74%) | 467 (39.95%) | 28 (60.87%) |  |
| Diabetes | 1,215 |  |  |  | 0.088 |
| No |  | 926 (76.21%) | 894 (76.48%) | 32 (69.57%) |  |
| Yes |  | 142 (11.69%) | 132 (11.29%) | 10 (21.74%) |  |
| Borderline |  | 147 (12. 10%) | 143 (12.23%) | 4 (8.70%) |  |
| Citrus, Melons, and Berries a, Mean ± SD | 1,215 | 0.19 ± 0.66 | 0.19 ± 0.67 | 0.08 ± 0.21 | 0.088 |
| Other Intact Fruits a, Mean ± SD | 1,215 | 0.43 ± 0.65 | 0.43 ± 0.65 | 0.43 ± 0.66 | 0.559 |
| Fruit Juices a, Mean ± SD | 1,215 | 0.27 ± 0.65 | 0.27 ± 0.64 | 0.34 ± 0.87 | 0.948 |
| Total Fruits a, Mean ± SD | 1,215 | 0.89 ± 1.24 | 0.89 ± 1.24 | 0.86 ± 1.15 | 0.671 |
| Dark Green Vegetables a, Mean ± SD | 1,215 | 0.13 ± 0.31 | 0.13 ± 0.31 | 0.10 ± 0.27 | 0.221 |
| Tomatoes a, Mean ± SD | 1,215 | 0.26 ± 0.31 | 0.25 ± 0.31 | 0.28 ± 0.37 | 0.865 |
| Other Red and Orange  Vegetables a, Mean ± SD | 1,215 | 0.07 ± 0.15 | 0.07 ± 0.15 | 0.07 ± 0.19 | 0.457 |
| Total Red and Orange  Vegetables a, Mean ± SD | 1,215 | 0.33 ± 0.35 | 0.33 ± 0.35 | 0.35 ± 0.41 | 0.710 |
| White Potatoes a, Mean ± SD | 1,215 | 0.33 ± 0.46 | 0.33 ± 0.47 | 0.23 ± 0.28 | 0.543 |
| Other Starchy Vegetables a, Mean ± SD | 1,215 | 0.07 ± 0.17 | 0.07 ± 0.17 | 0.04 ± 0.08 | 0.406 |
| Total Starchy Vegetables a, Mean ± SD | 1,215 | 0.39 ± 0.51 | 0.40 ± 0.52 | 0.27 ± 0.31 | 0.220 |
| Other Vegetables a, Mean ± SD | 1,215 | 0.48 ± 0.53 | 0.48 ± 0.53 | 0.43 ± 0.38 | 0.846 |
| Total Vegetables a, Mean ± SD | 1,215 | 1.34 ± 0.99 | 1.34 ± 1.00 | 1.16 ± 0.79 | 0.332 |
| Legumes as Vegetables a, Mean ± SD | 1,215 | 0.10 ± 0.25 | 0.10 ± 0.25 | 0.12 ± 0.28 | 0.820 |
| Whole Grains a, Mean ± SD | 1,215 | 0.85 ± 1.19 | 0.85 ± 1.20 | 0.76 ± 1.02 | 0.652 |

| **Characteristic** | **N** | **Overall**  **N = 1,215** | **No Psoriasis**  **N = 1,169** | **Psoriasis**  **N = 46** | **p-value** |
| --- | --- | --- | --- | --- | --- |
| Refined Grains a, Mean ± SD | 1,215 | 4.96 ± 3.18 | 4.94 ± 3.18 | 5.25 ± 3.18 | 0.525 |
| Total Grains a, Mean ± SD | 1,215 | 5.80 ± 3.45 | 5.79 ± 3.45 | 6.01 ± 3.40 | 0.740 |
| Meat a, Mean ± SD | 1,215 | 1.47 ± 1.88 | 1.48 ± 1.88 | 1.35 ± 1.68 | 0.727 |
| Cured Meat a, Mean ± SD | 1,215 | 0.90 ± 1.24 | 0.90 ± 1.24 | 0.90 ± 1.08 | 0.719 |
| Organ Meat a, Mean ± SD | 1,215 | 0.02 ± 0.20 | 0.02 ± 0.20 | 0.02 ± 0.14 | 0.811 |
| Poultry a, Mean ± SD | 1,215 | 1.24 ± 1.72 | 1.26 ± 1.74 | 0.86 ± 1.21 | 0.152 |
| Seafood High in n-3 a, Mean ± SD | 1,215 | 0.14 ± 0.61 | 0.15 ± 0.62 | 0.06 ± 0.22 | 0.793 |
| Seafood Low in n-3 a, Mean ± SD | 1,215 | 0.39 ± 1.05 | 0.39 ± 1.06 | 0.26 ± 0.71 | 0.531 |
| Total Meat Poultry Seafood a, Mean ± SD | 1,215 | 4.17 ± 3.00 | 4.20 ± 3.03 | 3.45 ± 2.13 | 0.294 |
| Eggs a, Mean ± SD | 1,215 | 0.45 ± 0.65 | 0.45 ± 0.64 | 0.61 ± 0.71 | 0.113 |
| Soy Products a, Mean ± SD | 1,215 | 0.07 ± 0.30 | 0.07 ± 0.29 | 0.07 ± 0.44 | 0.043 |
| Nuts and Seeds a, Mean ± SD | 1,215 | 0.68 ± 1.63 | 0.68 ± 1.63 | 0.78 ± 1.66 | 0.387 |
| Legumes as Protein Foods a, Mean ± SD | 1,215 | 0.39 ± 1.00 | 0.38 ± 0.99 | 0.47 ± 1.13 | 0.835 |
| Total Protein Foods a, Mean ± SD | 1,215 | 5.38 ± 3.56 | 5.40 ± 3.59 | 4.90 ± 2.69 | 0.700 |
| Milk a, Mean ± SD | 1,215 | 0.74 ± 0.94 | 0.74 ± 0.92 | 0.84 ± 1.27 | 0.665 |
| Yogurt a, Mean ± SD | 1,215 | 0.06 ± 0.19 | 0.07 ± 0.19 | 0.02 ± 0.09 | 0.030 |
| Cheese a, Mean ± SD | 1,215 | 0.71 ± 0.89 | 0.70 ± 0.89 | 0.90 ± 0.89 | 0.091 |
| Total Dairy a, Mean ± SD | 1,215 | 1.55 ± 1.34 | 1.54 ± 1.34 | 1.78 ± 1.49 | 0.322 |
| Oils a, Mean ± SD | 1,215 | 21.69 ± 16.99 | 21.68 ± 16.97 | 22.02 ± 17.83 | 0.822 |
| Solid Fats a, Mean ± SD | 1,215 | 34.05 ± 22.14 | 33.98 ± 22.27 | 36.03 ± 18.61 | 0.164 |
| Added Sugars a, Mean ± SD | 1,215 | 17.48 ± 15.17 | 17.47 ± 15.08 | 17.73 ± 17.46 | 0.770 |
| Alcoholic Beverages a, Mean ± | 1,215 | 0.60 ± 1.42 | 0.58 ± 1.31 | 1.10 ± 3.10 | 0.710 |

**Characteristic**

**N**

**Overall N = 1,215**

**No Psoriasis N = 1,169**

**Psoriasis N = 46**

**p-value**

SD

a: Student t-test, b: Chi-square test, SD: standard deviation

Supplementary Table 2. Food Group Intake by Psoriasis Status – MyPyramid Equivalents Database Note: Intake levels are expressed as mean ± SD. Statistical comparisons were performed using Student’s t-test. Abbreviations: SD, standard deviation.

| **Characteristic** | **N** | **Overall**  **N = 1257** | **No Psoriasis**  **N = 1211** | **Psoriasis**  **N = 46** | **p-value** |
| --- | --- | --- | --- | --- | --- |

| Age a, Mean ± SD | 1257 | 49.20 ± 15.54 | 49.13 ± 15.53 | 50.83 ± 16.06 | 0.469 |
| --- | --- | --- | --- | --- | --- |
| Sex b, n(%) | 1257 |  |  |  | 0.931 |
| FeMale | 1257 | 800 (63.64) 771 (63.67) | | 29 (63.04) |  |
| Male | 1257 | 457 (36.36) 440 (36.33) | | 17 (36.96) |  |
| Race/ethnicity b, n(%) | 1257 |  |  |  | 0.243 |
| Mexican | 1257 | 99 (7.88) 93 (7.68) | | 6 (13.04) |  |
| Other Hispanic | 1257 | 75 (5.97) 72 (5.95) | | 3 (6.52) |  |
| Non-Hispanic White | 1257 | 845 (67.22) 816 (67.38) | | 29 (63.04) |  |
| Non-Hispanic Black | 1257 | 160 (12.73) 157 (12.96) | | 3 (6.52) |  |
| Other Race | 1257 | 78 (6.21) 73 (6.03) | | 5 (10.87) |  |
| Education b, n(%) | 1257 |  |  |  | 0.580 |
| <9th grade | 1257 | 66 (5.25) 63 (5.20) | | 3 (6.52) |  |
| 9–11th grade | 1257 | 158 (12.57) 152 (12.55) | | 6 (13.04) |  |
| High school diploma/GED | 1257 | 296 (23.55) 281 (23.20) | | 15 (32.61) |  |
| Some College/AA degree | 1257 | 428 (34.05) 416 (34.35) | | 12 (26.09) |  |
| ≥College graduate | 1257 | 309 (24.58) 299 (24.69) | | 10 (21.74) |  |
| Smoking status b, n(%) | 1257 |  |  |  | 0.859 |
| No | 1257 | 835 (66.43) 805 (66.47) | | 30 (65.22) |  |
| Yes | 1257 | 422 (33.57) 406 (33.53) | | 16 (34.78) |  |

| **Characteristic** | **N** | **Overall**  **N = 1257** | **No Psoriasis**  **N = 1211** | **Psoriasis**  **N = 46** | **p-value** |
| --- | --- | --- | --- | --- | --- |
| Drinking status b, n(%) | 1257 |  |  |  | 1.000 |
| No | 1257 | 122 (9.71) | 118 (9.74) | 4 (8.70) |  |
| Yes | 1257 | 1135 (90.29) | 1093 (90.26) | 42 (91.30) |  |
| Hypertension b, n(%) | 1257 |  |  |  | **0.005** |
| No | 1257 | 745 (59.27) | 727 (60.03) | 18 (39. 13) |  |
| Yes | 1257 | 512 (40.73) | 484 (39.97) | 28 (60.87) |  |
| Diabetes b, n(%) | 1257 |  |  |  | 0.099 |
| No | 1257 | 959 (76.29) | 927 (76.55) | 32 (69.57) |  |
| Yes | 1257 | 149 (11.85) | 139 (11.48) | 10 (21.74) |  |
| Borderline | 1257 | 149 (11.85) | 145 (11.97) | 4 (8.70) |  |
| Sport and energy drinks a, Mean ± SD | 1257 | 15.21 ±  112.30 | 15.79 ±  114.37 | 0.00 ± 0.00 | 0.349 |
| Fruit drinks a, Mean ± SD | 1257 | 53.07 ±  169.42 | 53.52 ±  171.30 | 41.35 ±  109.35 | 0.633 |
| Soft drinks a, Mean ± SD | 1257 | 216.27 ± 440.79 | 216.16 ± 440.85 | 219.29 ± 444.18 | 0.962 |
| Other diet drinks a, Mean ± SD | 1257 | 18.23 ±  159.95 | 18.28 ±  162.18 | 16.96 ± 82.62 | 0.956 |
| Diet soft drinks a, Mean ± SD | 1257 | 156.99 ± 417.87 | 156.73 ± 420.60 | 163.77 ± 341.82 | 0.911 |
| Vegetable juice a, Mean ± SD | 1257 | 4.33 ± 31.78 | 4.39 ± 32.19 | 2.63 ± 17.84 | 0.712 |
| Other fruit juice a, Mean ± SD | 1257 | 19.32 ± 99.19 | 19.58 ±  100.30 | 12.33 ± 63.65 | 0.626 |
| Apple juice a, Mean ± SD | 1257 | 6.40 ± 36.22 | 6.23 ± 35.90 | 10.78 ± 43.95 | 0.403 |
| Citrus juice a, Mean ± SD | 1257 | 34.14 ±  107.08 | 33.75 ±  104.57 | 44.52 ±  160.83 | 0.503 |
| Mashed potatoes and white potato mixtures a, Mean ± SD | 1257 | 17.80 ± 48.05 | 18.00 ± 48.50 | 12.48 ± 34.06 | 0.444 |
| French fries and other fried white potatoes a, Mean ± SD | 1257 | 14.46 ± 37.83 | 14.65 ± 38.36 | 9.29 ± 18.45 | 0.346 |
| White potatoes, baked or boiled a, Mean ± SD | 1257 | 5.36 ± 25.72 | 5.19 ± 25.42 | 9.67 ± 32.67 | 0.247 |
| Vegetable mixed dishes a, Mean ± SD | 1257 | 7.03 ± 33.41 | 7.19 ± 33.92 | 2.80 ± 13.67 | 0.382 |
| Other vegetables and combinations | 1257 | 16.78 ± | 16.93 ± | 12.85 ± | 0.517 |

| **Characteristic** | **N** | **Overall** | **No Psoriasis** | **Psoriasis**  **N = 46** | **p-value** |
| --- | --- | --- | --- | --- | --- |
|  |  | **N = 1257** | **N = 1211** |  |  |
| a, Mean ± SD |  | 41.94 | 42.30 | 31.19 |  |
| Other starchy vegetables a, Mean ± SD | 1257 | 2.22 ± 12.67 | 2.31 ± 12.90 | 0.00 ± 0.00 | 0.226 |
| Corn a, Mean ± SD | 1257 | 4.66 ± 19.17 | 4.73 ± 19.42 | 2.71 ± 10.84 | 0.483 |
| Onions a, Mean ± SD | 1257 | 2.91 ± 9.73 | 2.95 ± 9.85 | 1.80 ± 5.56 | 0.428 |
| String beans a, Mean ± SD | 1257 | 5.01 ± 17.96 | 4.90 ± 17.69 | 7.75 ± 24.13 | 0.291 |
| Lettuce and lettuce salads a, Mean ± SD | 1257 | 13.01 ± 31.28 | 13.25 ± 31.71 | 6.68 ± 15.00 | 0.162 |
| Dark green vegetables, excludes lettuce a, Mean ± SD | 1257 | 8.13 ± 28.22 | 8.20 ± 28.23 | 6.35 ± 28.17 | 0.663 |
| Other red and orange vegetables a, Mean ± SD | 1257 | 1.44 ± 9.69 | 1.47 ± 9.83 | 0.65 ± 4.39 | 0.570 |
| Carrots, Mean ± SD | 1257 | 3.33 ± 12.66 | 3.28 ± 12.09 | 4.58 ± 23.25 | 0.492 |
| Tomatoes a, Mean ± SD | 1257 | 11.73 ± 30.21 | 11.49 ± 29.69 | 17.99 ± 41.64 | 0.152 |
| Other fruits and fruit salads a, Mean ± SD | 1257 | 10.16 ± 34.64 | 10.29 ± 35.01 | 6.60 ± 23.15 | 0.478 |
| Dried fruits a, Mean ± SD | 1257 | 1.02 ± 6.82 | 1.03 ± 6.90 | 0.75 ± 4.16 | 0.782 |
| Melons a, Mean ± SD | 1257 | 11.73 ± 87.60 | 12.18 ± 89.21 | 0.00 ± 0.00 | 0.355 |
| Citrus fruits a, Mean ± SD | 1257 | 7.65 ± 30.61 | 7.69 ± 30.73 | 6.54 ± 27.56 | 0.803 |
| Berries a, Mean ± SD | 1257 | 6.93 ± 26.13 | 7.01 ± 26.26 | 4.74 ± 22.48 | 0.563 |
| Peaches and nectarines a, Mean ± SD | 1257 | 5.08 ± 30.73 | 4.87 ± 29.66 | 10.59 ± 51.60 | 0.216 |
| Grapes a, Mean ± SD | 1257 | 4.77 ± 25.48 | 4.78 ± 25.70 | 4.35 ± 18.89 | 0.909 |
| Bananas a, Mean ± SD | 1257 | 17.69 ± 38.27 | 17.94 ± 38.51 | 11.08 ± 30.82 | 0.233 |
| Apples a, Mean ± SD | 1257 | 16.75 ± 48.59 | 16.53 ± 48.35 | 22.46 ± 54.81 | 0.417 |
| Gelatins, ices, sorbets a, Mean ± SD | 1257 | 3.44 ± 21.75 | 3.19 ± 20.96 | 9.93 ± 36.82 | 0.224 |
| Pudding a, Mean ± SD | 1257 | 3.06 ± 19.73 | 3.12 ± 20.01 | 1.42 ± 9.62 | 0.565 |
| Ice cream and frozen dairy desserts a, Mean ± SD | 1257 | 21.06 ± 57.79 | 21.56 ± 58.62 | 7.94 ± 25.28 | 0.117 |
| Candy not containing chocolate a, Mean ± SD | 1257 | 5.00 ± 20.28 | 5.09 ± 20.61 | 2.56 ± 6.56 | 0.406 |

| **Characteristic** | **N** | **Overall** | **No Psoriasis** | **Psoriasis**  **N = 46** | **p-value** |
| --- | --- | --- | --- | --- | --- |
|  |  | **N = 1257** | **N = 1211** |  |  |
| Candy containing chocolate a, Mean ± SD | 1257 | 5.10 ± 14.11 | 5.19 ± 14.24 | 2.90 ± 9.94 | 0.282 |
| Doughnuts, sweet rolls, pastries a, Mean ± SD | 1257 | 7.66 ± 24.02 | 7.78 ± 24.32 | 4.33 ± 13.53 | 0.339 |
| Cookies and brownies a, Mean ± SD | 1257 | 9.64 ± 21.42 | 9.81 ± 21.69 | 5.23 ± 11.78 | 0.155 |
| Cakes and pies a, Mean ± SD | 1257 | 14.91 ± 43.34 | 14.98 ± 43.64 | 12.91 ± 34.72 | 0.750 |
| Nutrition bars a, Mean ± SD | 1257 | 0.49 ± 4.66 | 0.51 ± 4.74 | 0.00 ± 0.00 | 0.466 |
| Cereal bars a, Mean ± SD | 1257 | 0.78 ± 5.08 | 0.79 ± 5.15 | 0.40 ± 2.73 | 0.607 |
| Saltine crackers a, Mean ± SD | 1257 | 0.95 ± 4.09 | 0.94 ± 4.12 | 1.27 ± 3.38 | 0.588 |
| Crackers, excludes saltines a, Mean ± SD | 1257 | 3.79 ± 11.07 | 3.85 ± 11.22 | 2.13 ± 5.72 | 0.301 |
| Pretzels/snack mix a, Mean ± SD | 1257 | 1.87 ± 8.53 | 1.86 ± 8.54 | 2.01 ± 8.23 | 0.907 |
| Popcorn a, Mean ± SD | 1257 | 1.86 ± 8.66 | 1.93 ± 8.82 | 0.00 ± 0.00 | 0.137 |
| Tortilla, corn, other chips a, Mean ± SD | 1257 | 5.14 ± 16.31 | 5.04 ± 16.12 | 7.60 ± 20.66 | 0.297 |
| Potato chips a, Mean ± SD | 1257 | 1.40 ± 6.56 | 1.41 ± 6.57 | 1.06 ± 6.32 | 0.721 |
| Grits and other cooked cereals a, Mean ± SD | 1257 | 6.34 ± 47.12 | 6.45 ± 47.79 | 3.45 ± 23.42 | 0.672 |
| Oatmeal a, Mean ± SD | 1257 | 12.11 ± 53.18 | 12.24 ± 53.33 | 8.60 ± 49.40 | 0.649 |
| Ready-to-eat cereal, lower sugar (=<21 2g/100g) a, Mean ± SD | 1257 | 5.45 ± 14.93 | 5.34 ± 14.61 | 8.13 ± 21.83 | 0.214 |
| Ready-to-eat cereal, higher sugar (>21 2g/100g) a, Mean ± SD | 1257 | 7.50 ± 23.08 | 7.56 ± 23.26 | 5.90 ± 17.91 | 0.633 |
| Pancakes, waffles, French toast a, Mean ± SD | 1257 | 4.65 ± 18.91 | 4.73 ± 19.10 | 2.57 ± 12.81 | 0.445 |
| Biscuits, muffins, quick breads a, Mean ± SD | 1257 | 8.24 ± 34.09 | 8.26 ± 34.31 | 7.77 ± 27.70 | 0.924 |
| Tortillas a, Mean ± SD | 1257 | 4.66 ± 21.88 | 4.40 ± 21.22 | 11.32 ± 34.68 | 0.186 |
| Bagels and English muffins a, Mean ± SD | 1257 | 3.93 ± 15.17 | 3.77 ± 14.77 | 8.12 ± 23.18 | 0.056 |
| Rolls and buns a, Mean ± SD | 1257 | 8.24 ± 17.79 | 8.24 ± 17.80 | 8.27 ± 17.58 | 0.992 |
| Yeast breads a, Mean ± SD | 1257 | 27.21 ± 35 56 | 27.08 ± 35 51 | 30.83 ± 36 99 | 0.483 |

| **Characteristic** | **N** | **Overall** | **No Psoriasis** | **Psoriasis**  **N = 46** | **p-value** |
| --- | --- | --- | --- | --- | --- |
|  |  | **N = 1257** | **N = 1211** |  |  |
| Pasta, noodles a, cooked grains a, Mean ± SD | 1257 | 4.90 ± 24.67 | 4.93 ± 24.81 | 3.96 ± 20.99 | 0.793 |
| Rice a, Mean ± SD | 1257 | 6.07 ± 34.02 | 6.27 ± 34.63 | 0.86 ± 5.82 | 0.290 |
| Soups a, Mean ± SD | 1257 | 49.44 ±  132.44 | 48.68 ±  129.84 | 69.54 ±  189.46 | 0.295 |
| Other sandwiches a, Mean ± SD | 1257 | 5.29 ± 29.01 | 5.49 ± 29.54 | 0.00 ± 0.00 | 0.208 |
| Egg/breakfast sandwiches a, Mean ± SD | 1257 | 3.02 ± 18.50 | 2.68 ± 16.88 | 12.00 ± 42.47 | 0.145 |
| Chicken/turkey sandwiches a, Mean ± SD | 1257 | 5.17 ± 28.43 | 5.31 ± 28.89 | 1.55 ± 10.54 | 0.380 |
| Burgers a, Mean ± SD | 1257 | 8.43 ± 35.15 | 8.44 ± 35.32 | 7.99 ± 30.86 | 0.932 |
| Pizza a, Mean ± SD | 1257 | 24.17 ± 77.16 | 24.55 ± 78.14 | 14.30 ± 43.60 | 0.377 |
| Other Mexican mixed dishes a, Mean ± SD | 1257 | 5.94 ± 29.83 | 5.66 ± 28.83 | 13.49 ± 49.36 | 0.080 |
| Nachos a, Mean ± SD | 1257 | 0.62 ± 8.86 | 0.53 ± 8.21 | 2.85 ± 19.31 | 0.082 |
| Burritos and tacos a, Mean ± SD | 1257 | 8.52 ± 43.14 | 8.42 ± 43.34 | 11.10 ± 37.55 | 0.680 |
| Egg rolls, dumplings, sushi a, Mean ± SD | 1257 | 2.02 ± 15.37 | 2.02 ± 15.60 | 2.01 ± 7.28 | 0.993 |
| Stir-fry and soy-based sauce mixtures a, Mean ± SD | 1257 | 6.43 ± 33.53 | 6.39 ± 33.56 | 7.26 ± 33.05 | 0.863 |
| Fried rice and lo/chow mein a, Mean ± SD | 1257 | 5.56 ± 46.12 | 5.59 ± 46.58 | 4.76 ± 32.25 | 0.904 |
| Turnovers and other grain-based items a, Mean ± SD | 1257 | 3.10 ± 20.41 | 3.14 ± 20.65 | 1.90 ± 12.90 | 0.686 |
| Macaroni and cheese a, Mean ± SD | 1257 | 8.64 ± 38.58 | 8.51 ± 38.33 | 12.17 ± 44.86 | 0.528 |
| Pasta mixed dishes, excludes  macaroni and cheese a, Mean ± SD | 1257 | 25.21 ± 80.24 | 25.52 ± 80.36 | 17.01 ± 77.29 | 0.480 |
| Rice mixed dishes a, Mean ± SD | 1257 | 7.73 ± 40.67 | 7.85 ± 41.22 | 4.51 ± 21.72 | 0.585 |
| Seafood mixed dishes a, Mean ± SD | 1257 | 6.09 ± 40.20 | 6.11 ± 40.78 | 5.65 ± 19.80 | 0.939 |
| Poultry mixed dishes a, Mean ± SD | 1257 | 17.33 ± 55.34 | 17.42 ± 55.33 | 14.87 ± 56.15 | 0.759 |
| Meat mixed dishes a, Mean ± SD | 1257 | 26.81 ± 75 84 | 26.90 ± 75 67 | 24.44 ± 80 97 | 0.829 |

| **Characteristic** | **N** | **Overall** | **No Psoriasis** | **Psoriasis**  **N = 46** | **p-value** |
| --- | --- | --- | --- | --- | --- |
|  |  | **N = 1257** | **N = 1211** |  |  |
| Processed soy products a, Mean ± SD | 1257 | 1.07 ± 10.59 | 1.12 ± 10.79 | 0.00 ± 0.00 | 0.483 |
| Nuts and seeds a, Mean ± SD | 1257 | 7.96 ± 23.56 | 7.82 ± 23.43 | 11.75 ± 26.79 | 0.266 |
| Beans, peas, legumes a, Mean ± SD | 1257 | 10.90 ± 38.80 | 10.72 ± 38.50 | 15.50 ± 46.25 | 0.412 |
| Sausages a, Mean ± SD | 1257 | 2.94 ± 16.57 | 2.93 ± 16.58 | 3.11 ± 16.49 | 0.945 |
| Frankfurters a, Mean ± SD | 1257 | 2.50 ± 14.65 | 2.46 ± 14.67 | 3.72 ± 14.23 | 0.567 |
| Bacon a, Mean ± SD | 1257 | 1.32 ± 4.71 | 1.36 ± 4.79 | 0.35 ± 1.40 | 0.152 |
| Cold cuts and cured meats a, Mean ± SD | 1257 | 13.88 ± 29.83 | 13.92 ± 30.06 | 12.79 ± 23.04 | 0.802 |
| Eggs and omelets a, Mean ± SD | 1257 | 8.20 ± 27.17 | 8.11 ± 27.23 | 10.63 ± 25.79 | 0.537 |
| Shellfish a, Mean ± SD | 1257 | 2.10 ± 12.37 | 2.15 ± 12.56 | 0.74 ± 5.01 | 0.447 |
| Fish a, Mean ± SD | 1257 | 10.32 ± 36.48 | 10.53 ± 37.01 | 4.76 ± 16.66 | 0.292 |
| Turkey, duck, other poultry a, Mean ± SD | 1257 | 2.72 ± 14.39 | 2.78 ± 14.58 | 1.16 ± 7.87 | 0.455 |
| Chicken patties, nuggets and tenders a, Mean ± SD | 1257 | 2.42 ± 13.71 | 2.42 ± 13.62 | 2.37 ± 16.10 | 0.981 |
| Chicken, whole pieces a, Mean ± SD | 1257 | 18.49 ± 42.31 | 18.55 ± 42.37 | 17.09 ± 41.18 | 0.819 |
| Liver and organ meats a, Mean ± SD | 1257 | 0.38 ± 4.98 | 0.40 ± 5.08 | 0.00 ± 0.00 | 0.594 |
| Lamb, goat, game a, Mean ± SD | 1257 | 1.32 ± 11.38 | 1.37 ± 11.59 | 0.00 ± 0.00 | 0.423 |
| Pork a, Mean ± SD | 1257 | 6.01 ± 21.92 | 6.06 ± 22.05 | 4.71 ± 18.12 | 0.682 |
| Ground beef a, Mean ± SD | 1257 | 8.15 ± 25.54 | 8.17 ± 25.70 | 7.81 ± 20.99 | 0.926 |
| Beef, excludes ground a, Mean ± SD | 1257 | 12.01 ± 34.29 | 12.08 ± 34.44 | 10.25 ± 30.41 | 0.723 |
| Yogurt, lowfat and nonfat a, Mean ± SD | 1257 | 11.38 ± 44.95 | 11.76 ± 45.72 | 1.23 ± 8.36 | 0.119 |
| Yogurt, whole and reduced fat a, Mean ± SD | 1257 | 0.92 ± 9.67 | 0.95 ± 9.85 | 0.00 ± 0.00 | 0.512 |
| Cottage/ricotta cheese a, Mean ± SD | 1257 | 2.76 ± 19.44 | 2.54 ± 18.63 | 8.62 ± 34.37 | 0.239 |
| Cheese a, Mean ± SD | 1257 | 10.35 ± 20 58 | 10.13 ± 20 19 | 16.03 ± 28 85 | 0.056 |

| **Characteristic** | **N** | **Overall** | **No Psoriasis** | **Psoriasis**  **N = 46** | **p-value** |
| --- | --- | --- | --- | --- | --- |
|  |  | **N = 1257** | **N = 1211** |  |  |
| Milk substitutes a, Mean ± SD | 1257 | 3.58 ± 29.33 | 3.69 ± 29.87 | 0.67 ± 4.52 | 0.493 |
| Milk shakes and other dairy drinks a, Mean ± SD | 1257 | 7.03 ± 45.43 | 6.51 ± 43.03 | 20.74 ± 87.17 | 0.276 |
| Flavored milk, lowfat a, Mean ± SD | 1257 | 2.10 ± 26.43 | 2.18 ± 26.92 | 0.00 ± 0.00 | 0.583 |
| Flavored milk, reduced fat a, Mean ± SD | 1257 | 2.92 ± 33.05 | 3.03 ± 33.67 | 0.00 ± 0.00 | 0.542 |
| Flavored milk, whole a, Mean ± SD | 1257 | 2.69 ± 39.39 | 2.42 ± 39.06 | 9.85 ± 46.97 | 0.209 |
| Milk, nonfat a, Mean ± SD | 1257 | 24.50 ±  105.12 | 24.41 ±  104.30 | 26.78 ±  126.10 | 0.881 |
| Milk, lowfat a, Mean ± SD | 1257 | 17.79 ± 78.80 | 17.54 ± 75.38 | 24.37 ±  143.09 | 0.565 |
| Milk, reduced fat a, Mean ± SD | 1257 | 47.22 ±  164.79 | 48.06 ±  167.25 | 24.97 ± 73.05 | 0.351 |
| Milk, whole a, Mean ± SD | 1257 | 26.34 ±  111.42 | 24.80 ±  102.53 | 66.86 ±  249.11 | 0.260 |
| Diet sport and energy drinks a, Mean ± SD | 1257 | 1.38 ± 28.75 | 1.44 ± 29.29 | 0.00 ± 0.00 | 0.739 |
| Flavored milk, nonfat a, Mean ± SD | 1257 | 0.44 ± 9.50 | 0.45 ± 9.68 | 0.00 ± 0.00 | 0.752 |

a: Student t-test, b: Chi-square test, SD: standard deviation

Supplementary Table 3. Specific Food and Beverage Intake by Psoriasis Status – WWEIA Database Note: Values represent mean daily intake (g) ± SD. p-values were calculated using Student’s t-test.

Abbreviations: SD, standard deviation.

Supplementary Material

**Supplementary Tables 4–10**

*Leakage-controlled cross-validation and locked-validation results.*

**Supplementary Table 4. Best tuned five-fold cross-validation performance for the Nutrient representation.**

| **model** | **parameters** | **cv roc auc mean** | **cv_roc_auc_sd** | **cv_pr_auc_mean** | **cv_pr_auc_sd** | **cv_brier_mean** | **cv_brier_sd** |
| --- | --- | --- | --- | --- | --- | --- | --- |
| Logistic regression | {"C": 1, "penalty":  "l2"} | 0.587 | 0.060 | 0.083 | 0.037 | 0.204 | 0.011 |
| SVM | {"C": 10,  "gamma": "scale",  "kernel": "rbf"} | 0.571 | 0.112 | 0.065 | 0.021 | 0.091 | 0.021 |
| Random forest | {"max_depth": 8,  "max_features":  0.5,  "min_samples_leaf  ": 3} | 0.548 | 0.110 | 0.057 | 0.017 | 0.133 | 0.019 |
| XGBoost | {"colsample_bytree  ": 0.8,  "learning_rate":  0.03, "max_depth":  2, "n_estimators":  100, "subsample":  0.8} | 0.512 | 0.076 | 0.054 | 0.012 | 0.160 | 0.015 |
| K-nearest  neighbors | {"n_neighbors":  21, "weights":  "distance"} | 0.502 | 0.127 | 0.052 | 0.019 | 0.331 | 0.036 |
| Naive Bayes | {"var_smoothing":  1e-09} | 0.474 | 0.127 | 0.046 | 0.013 | 0.195 | 0.054 |
| LightGBM | {"learning_rate":  0.03,  "min_child_sample  s": 10,  "n_estimators":  150, "num_leaves":  15} | 0.463 | 0.136 | 0.044 | 0.011 | 0.118 | 0.029 |

**Supplementary Table 5. Locked-validation performance for the Nutrient representation.**

| **model** | **best_paramet**  **ers** | **cv roc auc** | **ROC-AUC** | **AUC-PR** | **Brier** | **Accuracy** | **Sensitivity** | **Specificity** | **F1** |
| --- | --- | --- | --- | --- | --- | --- | --- | --- | --- |
| Logistic  regression | {"C": 1,  "penalty":  "l2"} | 0.587 | 0.645 | 0.063 | 0.176 | 0.666 | 0.545 | 0.670 | 0.105 |
| SVM | {"C": 10,  "gamma":  "scale",  "kernel":  "rbf"} | 0.571 | 0.673 | 0.067 | 0.107 | 0.430 | 0.727 | 0.418 | 0.084 |
| Random forest | {"max_depth":  8,  "max_features  ": 0.5,  "min_samples  _leaf": 3} | 0.548 | 0.684 | 0.071 | 0.133 | 0.328 | 0.909 | 0.306 | 0.089 |
| XGBoost | {"colsample_b  ytree": 0.8,  "learning_rate  ": 0.03,  "max_depth":  2,  "n_estimators"  : 100,  "subsample":  0.8} | 0.512 | 0.668 | 0.068 | 0.159 | 0.744 | 0.364 | 0.759 | 0.093 |
| K-nearest  neighbors | {"n_neighbors  ": 21,  "weights":  "distance"} | 0.502 | 0.709 | 0.112 | 0.362 | 0.793 | 0.364 | 0.810 | 0.113 |
| Naive Bayes | {"var_smoothi  ng": 1e-09} | 0.474 | 0.560 | 0.046 | 0.259 | 0.436 | 0.727 | 0.425 | 0.085 |
| LightGBM | {"learning_rat  e": 0.03,  "min_child_sa  mples": 10,  "n_estimators"  : 150,  "num_leaves":  15} | 0.463 | 0.634 | 0.053 | 0.119 | 0.275 | 0.909 | 0.252 | 0.083 |

**Supplementary Table 6. Best tuned five-fold cross-validation performance for the MPED representation.**

| **model** | **parameters** | **cv roc auc mean** | **cv_roc_auc_sd** | **cv_pr_auc_mean** | **cv_pr_auc_sd** | **cv_brier_mean** | **cv_brier_sd** |
| --- | --- | --- | --- | --- | --- | --- | --- |
| Logistic regression | {"C": 0.1,  "penalty": "l2"} | 0.563 | 0.114 | 0.082 | 0.059 | 0.223 | 0.018 |
| Random forest | {"max_depth": 8,  "max_features":  0.5,  "min_samples_leaf  ": 3} | 0.539 | 0.085 | 0.052 | 0.011 | 0.130 | 0.008 |
| XGBoost | {"colsample_bytree  ": 0.8,  "learning_rate":  0.03, "max_depth":  3, "n_estimators":  150, "subsample":  0.8} | 0.535 | 0.125 | 0.053 | 0.017 | 0.133 | 0.007 |
| K-nearest  neighbors | {"n_neighbors":  21, "weights":  "distance"} | 0.524 | 0.091 | 0.050 | 0.008 | 0.368 | 0.035 |
| SVM | {"C": 1, "gamma":  "scale", "kernel":  "rbf"} | 0.523 | 0.094 | 0.066 | 0.042 | 0.115 | 0.018 |
| LightGBM | {"learning_rate":  0.03,  "min_child_sample  s": 10,  "n_estimators":  150, "num_leaves":  15} | 0.503 | 0.076 | 0.046 | 0.007 | 0.119 | 0.019 |
| Naive Bayes | {"var_smoothing":  1e-09} | 0.503 | 0.115 | 0.055 | 0.022 | 0.341 | 0.078 |

**Supplementary Table 7. Locked-validation performance for the MPED representation.**

| **model** | **best_paramet**  **ers** | **cv roc auc** | **ROC-AUC** | **AUC-PR** | **Brier** | **Accuracy** | **Sensitivity** | **Specificity** | **F1** |
| --- | --- | --- | --- | --- | --- | --- | --- | --- | --- |
| Logistic  regression | {"C": 0.1,  "penalty":  "l2"} | 0.563 | 0.396 | 0.032 | 0.252 | 0.419 | 0.571 | 0.413 | 0.070 |
| Random forest | {"max_depth":  8,  "max_features  ": 0.5,  "min_samples  _leaf": 3} | 0.539 | 0.333 | 0.031 | 0.164 | 0.627 | 0.143 | 0.647 | 0.029 |
| XGBoost | {"colsample_b  ytree": 0.8,  "learning_rate  ": 0.03,  "max_depth":  3,  "n_estimators"  : 150,  "subsample":  0.8} | 0.535 | 0.410 | 0.033 | 0.173 | 0.586 | 0.286 | 0.598 | 0.050 |
| K-nearest  neighbors | {"n_neighbors  ": 21,  "weights":  "distance"} | 0.524 | 0.362 | 0.031 | 0.433 | 0.200 | 0.643 | 0.182 | 0.058 |
| SVM | {"C": 1,  "gamma":  "scale",  "kernel":  "rbf"} | 0.523 | 0.453 | 0.035 | 0.149 | 0.268 | 0.786 | 0.248 | 0.076 |
| LightGBM | {"learning_rat  e": 0.03,  "min_child_sa  mples": 10,  "n_estimators"  : 150,  "num_leaves":  15} | 0.503 | 0.365 | 0.034 | 0.145 | 0.447 | 0.214 | 0.456 | 0.029 |
| Naive Bayes | {"var_smoothi  ng": 1e-09} | 0.503 | 0.406 | 0.032 | 0.391 | 0.449 | 0.500 | 0.447 | 0.065 |

**Supplementary Table 8. Best tuned five-fold cross-validation performance for the WWEIA representation.**

| **model** | **parameters** | **cv roc auc mean** | **cv_roc_auc_sd** | **cv_pr_auc_mean** | **cv_pr_auc_sd** | **cv_brier_mean** | **cv_brier_sd** |
| --- | --- | --- | --- | --- | --- | --- | --- |
| XGBoost | {"colsample_bytree  ": 0.8,  "learning_rate":  0.03, "max_depth":  2, "n_estimators":  100, "subsample":  0.8} | 0.560 | 0.049 | 0.085 | 0.055 | 0.132 | 0.007 |
| Random forest | {"max_depth": 8,  "max_features":  0.5,  "min_samples_leaf  ": 3} | 0.552 | 0.069 | 0.088 | 0.040 | 0.109 | 0.010 |
| LightGBM | {"learning_rate":  0.03,  "min_child_sample  s": 10,  "n_estimators":  100, "num_leaves":  7} | 0.537 | 0.100 | 0.080 | 0.043 | 0.120 | 0.008 |
| K-nearest  neighbors | {"n_neighbors":  21, "weights":  "distance"} | 0.514 | 0.094 | 0.075 | 0.061 | 0.300 | 0.040 |
| SVM | {"C": 10,  "gamma": "scale",  "kernel": "rbf"} | 0.498 | 0.067 | 0.064 | 0.029 | 0.084 | 0.031 |
| Logistic regression | {"C": 0.1,  "penalty": "l2"} | 0.473 | 0.098 | 0.047 | 0.016 | 0.197 | 0.021 |
| Naive Bayes | {"var_smoothing":  1e-09} | 0.410 | 0.143 | 0.049 | 0.029 | 0.314 | 0.074 |

**Supplementary Table 9. Locked-validation performance for the WWEIA representation.**

| **model** | **best_paramet**  **ers** | **cv roc auc** | **ROC-AUC** | **AUC-PR** | **Brier** | **Accuracy** | **Sensitivity** | **Specificity** | **F1** |
| --- | --- | --- | --- | --- | --- | --- | --- | --- | --- |
| XGBoost | {"colsample_b  ytree": 0.8,  "learning_rate  ": 0.03,  "max_depth":  2,  "n_estimators"  : 100,  "subsample":  0.8} | 0.560 | 0.401 | 0.031 | 0.124 | 0.511 | 0.429 | 0.514 | 0.061 |
| Random forest | {"max_depth":  8,  "max_features  ": 0.5,  "min_samples  _leaf": 3} | 0.552 | 0.413 | 0.031 | 0.107 | 0.627 | 0.071 | 0.648 | 0.014 |
| LightGBM | {"learning_rat  e": 0.03,  "min_child_sa  mples": 10,  "n_estimators"  : 100,  "num_leaves":  7} | 0.537 | 0.316 | 0.027 | 0.108 | 0.913 | 0.000 | 0.948 | 0.000 |
| K-nearest  neighbors | {"n_neighbors  ": 21,  "weights":  "distance"} | 0.514 | 0.472 | 0.035 | 0.355 | 0.376 | 0.714 | 0.363 | 0.078 |
| SVM | {"C": 10,  "gamma":  "scale",  "kernel":  "rbf"} | 0.498 | 0.439 | 0.033 | 0.098 | 0.823 | 0.000 | 0.854 | 0.000 |
| Logistic  regression | {"C": 0.1,  "penalty":  "l2"} | 0.473 | 0.461 | 0.041 | 0.199 | 0.119 | 0.857 | 0.091 | 0.067 |
| Naive Bayes | {"var_smoothi  ng": 1e-09} | 0.410 | 0.482 | 0.037 | 0.342 | 0.836 | 0.000 | 0.868 | 0.000 |

**Supplementary Table 10. Hyperparameter search space and selected settings.**

| **dataset** | **model** | **tested_parameter_sets** | **selected_parameters** | **selected_cv_auc** |
| --- | --- | --- | --- | --- |
| MPED | K-nearest neighbors | {"n_neighbors": 11, "weights":  "distance"} \| {"n_neighbors": 21,  "weights": "distance"} | {"n_neighbors": 21, "weights":  "distance"} | 0.524 |

| MPED | LightGBM | {"learning_rate": 0.03,  "min_child_samples": 10,  "n_estimators": 100,  "num_leaves": 7} \|  {"learning_rate": 0.03,  "min_child_samples": 10,  "n_estimators": 150,  "num_leaves": 15} | {"learning_rate": 0.03,  "min_child_samples": 10,  "n_estimators": 150,  "num_leaves": 15} | 0.503 |
| --- | --- | --- | --- | --- |
| MPED | Logistic regression | {"C": 0.1, "penalty": "l2"} \| {"C":  1, "penalty": "l2"} | {"C": 0.1, "penalty": "l2"} | 0.563 |
| MPED | Naive Bayes | {"var_smoothing": 1e-09} \|  {"var_smoothing": 1e-05} | {"var_smoothing": 1e-09} | 0.503 |
| MPED | Random forest | {"max_depth": null,  "max_features": "sqrt",  "min_samples_leaf": 3} \|  {"max_depth": 8,  "max_features": 0.5,  "min_samples_leaf": 3} | {"max_depth": 8,  "max_features": 0.5, "min_samples_leaf": 3} | 0.539 |
| MPED | SVM | {"C": 1, "gamma": "scale", "kernel": "rbf"} \| {"C": 10, "gamma": "scale", "kernel":  "rbf"} | {"C": 1, "gamma": "scale", "kernel": "rbf"} | 0.523 |
| MPED | XGBoost | {"colsample_bytree": 0.8,  "learning_rate": 0.03,  "max_depth": 2, "n_estimators":  100, "subsample": 0.8} \|  {"colsample_bytree": 0.8,  "learning_rate": 0.03,  "max_depth": 3, "n_estimators":  150, "subsample": 0.8} | {"colsample_bytree": 0.8,  "learning_rate": 0.03,  "max_depth": 3, "n_estimators":  150, "subsample": 0.8} | 0.535 |
| Nutrient | K-nearest neighbors | {"n_neighbors": 11, "weights":  "distance"} \| {"n_neighbors": 21,  "weights": "distance"} | {"n_neighbors": 21, "weights":  "distance"} | 0.502 |
| Nutrient | LightGBM | {"learning_rate": 0.03,  "min_child_samples": 10,  "n_estimators": 100,  "num_leaves": 7} \|  {"learning_rate": 0.03,  "min_child_samples": 10,  "n_estimators": 150,  "num_leaves": 15} | {"learning_rate": 0.03,  "min_child_samples": 10,  "n_estimators": 150,  "num_leaves": 15} | 0.463 |
| Nutrient | Logistic regression | {"C": 0.1, "penalty": "l2"} \| {"C":  1, "penalty": "l2"} | {"C": 1, "penalty": "l2"} | 0.587 |
| Nutrient | Naive Bayes | {"var_smoothing": 1e-09} \|  {"var_smoothing": 1e-05} | {"var_smoothing": 1e-09} | 0.474 |
| Nutrient | Random forest | {"max_depth": null,  "max_features": "sqrt",  "min_samples_leaf": 3} \|  {"max_depth": 8,  "max_features": 0.5,  "min_samples_leaf": 3} | {"max_depth": 8,  "max_features": 0.5, "min_samples_leaf": 3} | 0.548 |
| Nutrient | SVM | {"C": 1, "gamma": "scale", "kernel": "rbf"} \| {"C": 10, "gamma": "scale", "kernel":  "rbf"} | {"C": 10, "gamma": "scale", "kernel": "rbf"} | 0.571 |
| Nutrient | XGBoost | {"colsample_bytree": 0.8,  "learning_rate": 0.03,  "max_depth": 2, "n_estimators":  100, "subsample": 0.8} \|  {"colsample_bytree": 0.8,  "learning_rate": 0.03,  "max_depth": 3, "n_estimators":  150, "subsample": 0.8} | {"colsample_bytree": 0.8,  "learning_rate": 0.03,  "max_depth": 2, "n_estimators":  100, "subsample": 0.8} | 0.512 |
| WWEIA | K-nearest neighbors | {"n_neighbors": 11, "weights":  "distance"} \| {"n_neighbors": 21,  "weights": "distance"} | {"n_neighbors": 21, "weights":  "distance"} | 0.514 |
| WWEIA | LightGBM | {"learning_rate": 0.03,  "min_child_samples": 10,  "n_estimators": 100,  "num_leaves": 7} \|  {"learning_rate": 0.03,  "min_child_samples": 10,  "n_estimators": 150,  "num_leaves": 15} | {"learning_rate": 0.03,  "min_child_samples": 10,  "n_estimators": 100,  "num_leaves": 7} | 0.537 |
| WWEIA | Logistic regression | {"C": 0.1, "penalty": "l2"} \| {"C":  1, "penalty": "l2"} | {"C": 0.1, "penalty": "l2"} | 0.473 |
| WWEIA | Naive Bayes | {"var_smoothing": 1e-09} \|  {"var_smoothing": 1e-05} | {"var_smoothing": 1e-09} | 0.410 |
| WWEIA | Random forest | {"max_depth": null,  "max_features": "sqrt",  "min_samples_leaf": 3} \|  {"max_depth": 8,  "max_features": 0.5,  "min_samples_leaf": 3} | {"max_depth": 8,  "max_features": 0.5, "min_samples_leaf": 3} | 0.552 |
| WWEIA | SVM | {"C": 1, "gamma": "scale", | {"C": 10, "gamma": "scale", | 0.498 |

|  |  | "kernel": "rbf"} \| {"C": 10, "gamma": "scale", "kernel":  "rbf"} | "kernel": "rbf"} |  |
| --- | --- | --- | --- | --- |
| WWEIA | XGBoost | {"colsample_bytree": 0.8,  "learning_rate": 0.03,  "max_depth": 2, "n_estimators":  100, "subsample": 0.8} \|  {"colsample_bytree": 0.8,  "learning_rate": 0.03,  "max_depth": 3, "n_estimators":  150, "subsample": 0.8} | {"colsample_bytree": 0.8,  "learning_rate": 0.03,  "max_depth": 2, "n_estimators":  100, "subsample": 0.8} | 0.560 |

**Notes.** All preprocessing and resampling steps were fitted within the training workflow; the 30% locked validation sets were not used for feature selection, class balancing, tuning, threshold selection, or model selection. The random seed was 20260830. Logistic regression was included as the conventional

multivariable benchmark requested by the reviewer.
